# Cancer prevention and screening for people with intellectual disabilities in Europe: a systematic review and recommendations for policy and practice

**DOI:** 10.3389/fpubh.2026.1925665

**Published:** 2026-08-20

**Authors:** Vladimir Vukovic, Peter Knapp, Kate Sykes, Margaret Denny, Oliwia Kowalczyk, Suzanne Denieffe, John Wells, Maarten Cuypers, Barrie Tyner, Kálya Yasmine Lima, Adela Elena Popa, Dalia Ismail Ibrahim, Rebecca Hansford, Trine Allerslev Horsbøl, Dragana Milutinović, Bilge Güvenç Tuna, Eda Başustaoğlu Şahin, Ayşegül Ilgaz, Amina Banda, Elif Sarac, Soner Doğan, Seda Cansu Yeniğün Akbulut, Bahar Aksoy, Dušanka Tadić, Mohammed A. Merzah, Pınar Soylar, Aysenur Doğan, Dragana Simin, Jasmin Mušanović, Damla Altin, Merve Sena Topkaya, Fatima Kassymbekova, Vladimir Trajkovski, Edanur Tar Bolacali, Louise Lynch, Eilish Burke, Martin McMahon

**Affiliations:** 1 Faculty of Medicine, University of Novi Sad, Novi Sad, Serbia; 2 Institute of Public Health of Vojvodina, Novi Sad, Serbia; 3 Department of Health Sciences and Hull York Medical School, University of York, York, United Kingdom; 4 School of Communities and Education, Faculty of Health and Wellbeing, Northumbria University, Newcastle upon Tyne, United Kingdom; 5 Faculty of Health Sciences, University of Maribor, Maribor, Slovenia; 6 Faculty of Health Sciences, Department of Oncology, Collegium Medicum Nicolaus Copernicus University, Toruń, Poland; 7 Institute of Advanced Studies, Nicolaus Copernicus University, Toruń, Poland; 8 Faculty of Arts and Humanities, South East Technological University, Waterford, Ireland; 9 South East Technological University, Waterford, Ireland; 10 Department of Primary and Community Care, Radboud University Medical Center, and Academic Collaborative Intellectual Disability and Health ‘Sterker op Eigen Benen’, Nijmegen, Netherlands; 11 Trinity Centre for Ageing and the Life Course in Intellectual Disability, School of Nursing and Midwifery, Trinity College Dublin, Dublin, Ireland; 12 Department of Social Work, Journalism, Public Relations and Sociology, Faculty of Social Sciences and Humanities, Lucian Blaga University of Sibiu, Sibiu, Romania; 13 Ministry of Health and Population, Cairo, Egypt; 14 School of Nursing, Queen’s University, Kingston, ON, Canada; 15 Division of Cancer Care and Epidemiology, Sinclair Cancer Research Institute, Queen’s University, Kingston, ON, Canada; 16 National Institute of Public Health, University of Southern Denmark, Copenhagen, Denmark; 17 Department of Nursing, Faculty of Medicine, University of Novi Sad, Novi Sad, Serbia; 18 Department of Biophysics, Faculty of Medicine, Yeditepe University, Istanbul, Türkiye; 19 Faculty of Health Sciences, Nursing Department, Giresun University, Giresun, Türkiye; 20 Department of Public Health Nursing, Faculty of Nursing, Akdeniz University, Antalya, Türkiye; 21 Ministry of Defense, Special Care Center for the Elderly, Ankara, Türkiye; 22 Department of Medical Biology, School of Medicine, Yeditepe University, Istanbul, Türkiye; 23 Kumluca Faculty of Health Sciences, Department of Surgical Disease Nursing, Akdeniz University, Antalya, Türkiye; 24 Kumluca Faculty of Health Sciences, Department of Child Health and Disease Nursing, Akdeniz University, Antalya, Türkiye; 25 Department of Nursing, College of Health Sciences, Academy of Applied Studies Belgrade, Belgrade, Serbia; 26 Department of Public Health and Epidemiology, Faculty of General Medicine, University of Debrecen, Debrecen, Hungary; 27 Department of Community Health Techniques, College of Polytechnic, Al-Furat Al-Awsat Technical University, Kufa, Iraq; 28 Department of Nursing, Faculty of Health Sciences, Fırat University, Elazığ, Türkiye; 29 Department of Medical Biology and Human Genetics, Faculty of Medicine, University of Sarajevo, Sarajevo, Bosnia and Herzegovina; 30 Department of Special Education, Faculty of Education, Sakarya University, Sakarya, Türkiye; 31 Department of Nutrition and Dietetics, Gülhane Health Sciences Institute, University of Health Sciences Türkiye, Ankara, Türkiye; 32 Asfendiyarov Kazakh National Medical University, Almaty, Kazakhstan; 33 Faculty of Philosophy, Institute of Special Education and Rehabilitation, Ss. Cyril and Methodius University, Skopje, North Macedonia; 34 Faculty of Health Sciences, Department of Child Health and Disease Nursing, Kırşehir Ahi Evran University, Kırşehir, Türkiye

**Keywords:** cancer, Europe, health inequalities, intellectual disability, policy, prevention, reasonable adjustments, screening

## Abstract

**Introduction:**

People with intellectual disability experience inequalities across the cancer care continuum, including lower screening participation, later-stage diagnosis, and higher cancer related mortality. Whether European cancer prevention and screening policies explicitly address this population has not been systematically mapped.

**Methods:**

This review aimed to identify and synthesise European national and international policy frameworks, guidelines, and recommendations for cancer prevention and screening that address this population. A systematic search of policy, recommendation and guideline documents across academic databases, grey literature portals, and organisational sources, to August 2025 was under taken. The Population, Concept, Context (PCC) framework guided eligibility. Two reviewers per country screened, extracted, and appraised documents indepen dently using the Authority, Accuracy, Coverage, Objectivity, Date, Significance (AACODS) checklist. Synthesis was narrative, stratified by cancer site and cancer control focus.

**Results:**

Sixteen documents met the inclusion criteria, originating from five jurisdictions: the United Kingdom, the Netherlands, Belgium, Denmark, and the European Union more broadly. Fifteen of the sixteen addressed cancer screen ing, one also addressed prevention, and two addressed clinical monitoring. Adjustments for people with intellectual disability were concentrated on breast, cervical, and bowel screening and grouped into accessible information, appoint ment adaptations, carer involvement, consent and capacity support, and work force training. Most documents originated from higher expenditure healthcare systems, and no eligible documents were retrieved from the majority of European Union member states.

**Discussion:**

Policy guidance for cancer prevention and screening for people with intellectual disability is unevenly distributed across Europe and con centrated in a small group of countries. Existing guidance should be extended beyond screening to encompass prevention, treatment, and survivorship, and EU coordination may help address this gap.

## 1 Introduction

Intellectual disability is a condition characterised by significant limitations in intellectual functioning and adaptive behaviour, with onset during the developmental period (1, 2). Prevalence is estimated at 1–3% of the general population, with more than 100 million people worldwide living with intellectual disability (3). People with intellectual disability are heterogeneous, and associated conditions such as epilepsy, higher chronic physical and mental health morbidity, and social and systemic barriers contribute to higher avoidable mortality and shorter life expectancy (4–8). Despite these elevated needs, people with intellectual disability remain underrepresented in public health monitoring, which narrows the evidence base for targeted health policy.

Evidence on cancer incidence and prevalence in adults with intellectual disability is mixed (9), whereas cancer outcomes consistently indicate disadvantage. A nationwide German study reported aggregate cancer prevalence of 4.2% compared with 5.1% in the general population (10), but site specific patterns are uneven. Brain cancer, leukaemia, and germ cell tumours occur more frequently, particularly in subgroups defined by genetic aetiology such as Down syndrome and PTEN related conditions (11, 12). The risk of testicular cancer in males with Down syndrome is elevated, with an estimated standardised incidence ratio over five times higher than the rate in the general population (SIR 5.5, 95% CI 1.8–13) (11). Other cancers are reported at lower aggregate rates in this population, although some of this may reflect underdiagnosis rather than reduced risk, as post-mortem studies have identified previously undiagnosed cancers (13).

Adults with intellectual disability are more likely to be diagnosed at advanced stages, including metastatic breast and colorectal cancers (14). Among those diagnosed with cancer, all-cause mortality is 1.49–2.74 times higher for adults with intellectual disability than for those without, and cancer specific mortality is similarly elevated (15). Cancer related mortality is approximately 1.5 times more likely to be a cause of death than in the general population (16) and cancers are also more likely to be diagnosed earlier in life than in the general population (17).

Cancer prevention and organised cancer screening reduce cancer burden through early detection and risk factor modification (18), and recent meta-analytic evidence links adherence to prevention guidelines with lower incidence across multiple cancer sites (19). Adults with intellectual disability participate less in population screening. In a recent Dutch study, they had 0.38 (95% CI 0.38–0.39), 0.40 (95% CI 0.39–0.40), and 0.42 (95% CI 0.41–0.42) times the odds of attending cervical, breast, and colorectal screening, respectively (20). Similarly, Danish population-based studies reported higher odds of never being screened among women with intellectual disability (OR = 4.90, 95% CI 4.60–5.22) (21), and reduced colorectal screening completion with a higher proportion of incomplete colonoscopies (22).

Barriers to participation operate at structural and individual levels. At the structural level, people with intellectual disability experience greater exposure to adverse social determinants of health, including reduced access to healthy foods and physical activity (23). Screening barriers include emotional discomfort, limited cancer and screening knowledge, inadequate appointment preparation, mobility and support needs, communication and consent difficulties, reliance on carers or support staff, and lack of accessible Easy-Read information (24, 25). Practical and organisational barriers, including scheduling, transport, complex healthcare systems, insufficiently adapted encounters, and negative interactions with undertrained professionals, operate throughout the pathway.

People with intellectual disability are under-recognised by cancer prevention and screening policies across Europe, with little to no inclusion in national strategies (26). This policy absence contributes to delayed detection and poorer outcomes and signals a need for unified policies at national and pan-European levels that acknowledge the healthcare needs of this group (27). Explicit inclusion of people with intellectual disability in cancer policy creates recognition and visibility, supports advocacy and accountability, and improves system planning. A cross-sectional survey of 29 organisations supporting people with intellectual disability across 14 European countries reported that few were aware of any national cancer prevention policy tailored to this population, and most that existed were considered inadequate (26).

Participation in cancer screening among adults with intellectual disability is shaped by modifiable organisational, professional, and contextual factors rather than by intellectual disability itself (24, 28). Common needs include accessible information, support from carers or advocates during screening appointments, and reasonable adjustments to standard screening procedures (24, 29, 30). Policies that fail to address these needs may exclude this population from screening programmes and prevention initiatives, even where no formal exclusion exists (26, 31). Without policy level direction, adjustments to screening services are likely to be inconsistent and unsupported by adequate guidance or resources (17, 32).

### 1.1 Rationale

A review of existing policy frameworks, guidelines, and recommendations on cancer prevention and screening for people with intellectual disability across Europe is needed to map current provision, identify gaps between what policies address and what this population requires, and provide a foundation for future policy development.

### 1.2 Objective

This study aimed to systematically identify, characterise, and synthesise European national, regional, and supranational policy frameworks, guidelines, and recommendations on cancer prevention and screening, with a focus on their inclusivity and specific adjustments for people with intellectual disability. The review quantified and categorised existing documents at the intersection of cancer prevention or screening and intellectual disability, and mapped gaps where specialised provisions for this population are lacking.

## 2 Methods

### 2.1 Data sources and search strategy

A combined academic database and grey literature search strategy identified eligible documents addressing cancer prevention or screening in people with intellectual disabilities up to August 2025. The academic database search covered PubMed/MEDLINE, Embase, Web of Science, PsycINFO, and CINAHL. Supplementary grey literature searches used Google Scholar, Policy Commons, structured Google searches of the first 100 results per country, and snowball searches of national databases and relevant organisations. Documents identified outside the initial searches were also included.

The strategy used the Population, Concept, Context (PCC) framework adapted for policy and grey literature review (33), plus a Country component for cross-national consistency:

- *Population:* People with intellectual disabilities, including those described in source documents as having learning disabilities or developmental disabilities.
- *Concept:* Cancer of any type or location. Prevention and screening terms were not used as search filters to avoid excluding documents of broader scope.
- *Context:* Publicly available documents intended to inform policy or practice, including policies, guidelines, legislation, recommendations, information leaflets, and strategic reports.
- *Country:* Searches were limited to the country of interest, i.e., 53 member states of the World Health Organisation European region, using country specific terms, including translation where required. The full UK search string is given below as an illustrative example. Per country strings are held by the country teams and available on request. The complete search strategy, including the search framework, information sources, and records retrieved by source, is provided in Supplementary Material (Search strategy).

Boolean operators, parentheses, and truncation optimised sensitivity and specificity. For example, the final UK search string, reviewed by an academic librarian, was:

(“learning disabil*” OR “intellectual disabil*” OR “developmental disabil*”) AND (cancer OR oncology OR carcinoma OR malign* OR tumor OR neoplasm*) AND (policy OR policies OR guidance* OR law* OR legal* OR guidelin* OR strateg* OR legislat* OR statutory OR governan* OR regulation* OR “health act*”) AND (“United Kingdom” OR “gov.uk” OR “nhs.uk” OR “nice.org.uk” OR “parliament.uk”)

### 2.2 Eligibility criteria

Documents were eligible regardless of publication year or country of origin, subject to the eligibility criteria below.

- *Document type:* Eligible documents comprised national or regional policies, clinical guidelines, recommendations, information leaflets, and legislative documents addressing cancer prevention or screening. Primary research studies were not eligible. The review focused specifically on policy and grey literature evidence base.
- *Population:* Documents were included if they addressed people with intellectual disabilities, with no age restrictions applied, including conditions within the intellectual disability umbrella. Uniform country search terms covered: intellectual disability, learning disability, developmental disability, Down syndrome, Fragile X syndrome, Williams syndrome, DiGeorge syndrome, 22q11 deletion syndrome, Prader–Willi syndrome, Angelman syndrome, Wolf–Hirschhorn syndrome, Cri du Chat syndrome, xeroderma pigmentosum, Fanconi anaemia, Nijmegen breakage syndrome, neurofibromatosis type 1, Bloom syndrome, Rubinstein–Taybi syndrome, Klippel–Trenaunay syndrome, Saethre–Chotzen syndrome, Cowden syndrome, Costello syndrome, Noonan syndrome, and disorders of sex development.
- *Topic:* Documents were eligible if they addressed cancer prevention (e.g., smoking cessation, healthy eating, physical activity and HPV vaccination) or cancer screening (e.g., cervical, breast, colorectal, prostate, and ovarian cancer screening). Documents addressing the prevention of avoidable deaths were also eligible where the factors examined were cancer related.
- *Reported content:* Documents were included only where the reported content related specifically to cancer in the population of interest, such as barriers or facilitators to access, accessibility of interventions, knowledge of cancer screening or prevention, motivation to engage, or interventions aimed at increasing cancer screening or prevention uptake.

### 2.3 Study selection and data extraction

In a multi-country process, one reviewer searched and screened each country, a second independently verified eligibility, and discrepancies were resolved by consensus.

Per country logs recorded reviewer identifiers, search dates, stage counts, the first 100 Google URLs, evidence of structured searches, and included documents. Duplicate removal used database native functions for indexed sources and manual country pair review for grey literature records, for which no consolidated deduplication log was retained. A PRISMA 2020 flow diagram summarises identification, screening, and inclusion across countries (34). The completed PRISMA 2020 checklist is provided in Supplementary Material.

Data extraction used a standardised Excel template covering document title, organisation, country and scope, publication date, cancer type, policy focus, aim, target audience, and content relating to people with intellectual disabilities. One reviewer extracted each document, with subset or full independent checking by the second country reviewer.

The primary outcome was identification and characterisation of policy frameworks, guidelines, recommendations, leaflets, and legislative documents addressing cancer prevention or screening in people with intellectual disabilities. All relevant content was extracted, with no restriction on cancer type, policy scope, or publication date.

### 2.4 Quality appraisal

The quality of the included documents was assessed using the Authority, Accuracy, Coverage, Objectivity, Date, Significance (AACODS) checklist for grey literature appraisal (35). Each document was independently evaluated by two reviewers across these six domains. Criteria were rated “Yes,” “No,” or “Unclear,” and discrepancies were resolved by consensus. The per document results are presented in Supplementary Table 2.

### 2.5 Synthesis

A narrative synthesis approach was used, consistent with the heterogeneity of the included documents and the approach set out in the registered review protocol. The documents included policies, guidelines, recommendations, leaflets, and legislative documents rather than primary quantitative studies, and variability in document type, scope, and reporting format precluded meta-analysis or quantitative pooling.

Documents were grouped along three axes for synthesis. First, by cancer site (breast, cervical, colorectal, prostate, ovarian, and other), to support comparison of site specific recommendations. Second, by cancer control focus (screening, prevention, treatment, clinical monitoring), to capture where each document operates on the cancer care continuum. Third, by geographical and administrative level (national, regional, supranational) and document type (policy, guideline, recommendation, leaflet, legislation), to characterise variation in scope and authority. Documents addressing multiple cancer sites were disaggregated into their site specific components and synthesised within each applicable category.

### 2.6 Use of an AI tool

After the human led searches and extraction had been completed in each country, ChatGPT (OpenAI; version and date of use given in Supplementary Material) was used by one author (VV) as a supplementary reliability cross-check on both the searches and the extraction. It searched online to identify any eligible documents the human led searches might have missed, and independently re-extracted data as a reliability check on the human led extraction. The tool informed no eligibility decisions and contributed no data to the synthesis, was not granted authorship, and was not relied on as a primary source. The full set of prompts is provided in the Supplementary Material. During the preparation of this manuscript the authors VV, BT & MM used Claude to refine, consolidate, and standardise the English language. After using this tool VV, BT, and MM reviewed and edited the content as needed and take full responsibility for the content of the publication.

### 2.7 Protocol registration and ethics

Protocol registration: The review protocol was registered on PROSPERO (registration number CRD420251140644). The review project is also available on the Open Science Framework.^1^

Ethics: This review did not involve primary data collection from human participants and therefore did not require institutional ethics approval. All included documents were publicly available at the time of extraction.

## 3 Results

### 3.1 Search results

**Figure 1:**
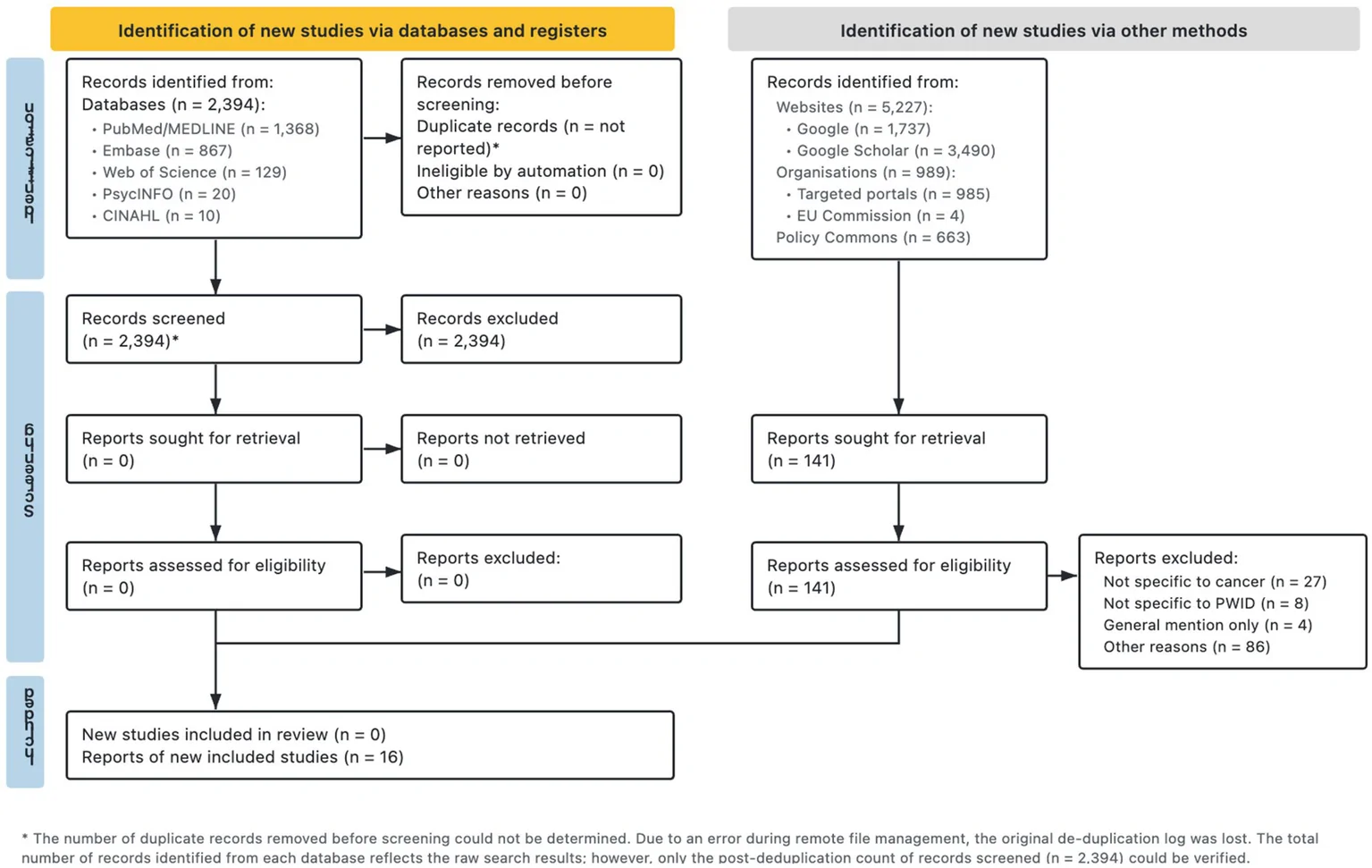
PRISMA 2020 flow diagram of database and grey literature searches.

The systematic database search, conducted across PubMed/MEDLINE, Embase, Web of Science, PsycINFO, and CINAHL, yielded a total of 2,394 records after deduplication. Duplicate counts were derived from each database’s native deduplication output. For grey literature sources, deduplication was managed via per country manual review without a consolidated log. The PRISMA flow diagram (Figure 1) gives full record counts. All database sourced records were excluded at title and abstract screening. The evidence base for this review derives entirely from grey literature sources (Google and snowball sampling).

Supplementary searching via grey literature methods identified an additional 6,879 records: websites (*n* = 5,227; Google, *n* = 1,737; Google Scholar, *n* = 3,490), organisational sources (*n* = 989; targeted portals, *n* = 985; European Commission, *n* = 4), and Policy Commons (*n* = 663). Given the volume and non-reproducibility of search engine results, Google screening was operationalised by restricting retrieval to the first 100 results per country per search date. PRISMA record counts for Google reflect records screened rather than total returned hits. Of the records identified through these supplementary methods, 141 reports were potentially eligible and sought for full-text retrieval. All 141 reports were successfully retrieved.

Full-text assessment of the 141 reports resulted in the exclusion of 125 documents for the following reasons: not specific to cancer (*n* = 27), not specific to people with intellectual disability (*n* = 8), general mention only without substantive content (*n* = 4), and other reasons (*n* = 86). A total of 16 documents met all inclusion criteria and were included in the review.

### 3.2 Characteristics of included documents

The 16 included documents originated from the UK (*n* = 9; England *n* = 7, Scotland *n* = 1, Wales *n* = 1), the Netherlands (*n* = 4), Belgium (*n* = 1), Denmark (*n* = 1), and the EU (*n* = 1). By document type, recommendations were most frequent (*n* = 9), followed by guidelines (*n* = 4) and policy documents (*n* = 3). Half of the documents were issued at national implementation level (*n* = 8). Fifteen of the 16 documents addressed cancer screening, with limited emphasis on prevention and clinical monitoring. Target audiences were varied, and methodological quality was high, with most documents scoring between 4 and 6 of 6 on the AACODS framework (Table 1).

**Table 1:** Characteristics of the included documents by country, with AACODS overall quality rating.

Country	Organisation	Year	Type	Level	Focus	Audience	AACODS
Belgium	Belgian Health Care Knowledge Centre (KCE) (49)	2022	Recommendation	National	Screening	Policymakers and health system planners, health services researchers, and stakeholders involved in health care access and policy for people with disabilities	6/6
Denmark	Region Hovedstaden – the regional health authority for the Capital Region in Denmark (51)	2025	Policy	Regional	Screening	Women eligible for cervical cancer screening; healthcare providers and general practitioners	4/6
European Union	European Commission, Joint Research Centre (JRC), Directorate F – Health, Consumers & Reference Materials, via the European Commission Initiative on Breast Cancer (ECIBC) (50)	2019	Recommendation	Supranational	Screening	Policymakers, public health programme designers, guideline developers	6/6
Netherlands	Nederlandse Vereniging van Artsen voor Verstandelijk Gehandicapten (NVAVG) (38)	2003	Guideline (clinical)	National	Screening	Physicians, GPs, disability care providers	6/6
Netherlands	Nederlandse Vereniging van Artsen voor Verstandelijk Gehandicapten (NVAVG) - the Dutch Association of Physicians for People with Intellectual Disabilities (47)	2004	Guideline (clinical)	National	Screening	GPs, physicians caring for women with intellectual disabilities, screening coordinators	6/6
Netherlands	Stichting Downsyndroom (Down Syndrome Foundation); Vereniging Samenwerkende Ouder- en Patiëntenorganisaties (VSOP); Nederlands Huisartsen Genootschap (NHG) - Dutch College of General Practitioners (40)	2016	Recommendation	National	Screening and clinical monitoring	General practitioners	6/6
Netherlands	Kinderneurologie.eu (41)	2026	Recommendation (informational/educational)	Regional	Clinical monitoring	Clinicians, families, caregivers	5/6
United Kingdom (England)	NHS Cancer Screening Programmes (48)	2009	Guideline	National	Screening	Healthcare professionals & screening programme staff	6/6
United Kingdom (England)	National Development Team for Inclusion (NDTi), in partnership with the Norah Fry Research Centre (University of Bristol) (43)	2013	Recommendation/practice guidance	Regional	Screening	Healthcare professionals, public health staff, screening teams.	6/6
United Kingdom (England)	Public Health England (46)	2016	Guideline (practice)	National	Screening	Healthcare professionals, carers, policy makers, families	6/6
United Kingdom (England)	North East and Cumbria Learning Disability Network (44)	2017	Recommendation (informational/educational)	Regional	Screening	Health and social care stakeholders, commissioners, clinicians	5/6
United Kingdom (England)	NHS England (45)	2017 (initial), updated 2025	Recommendation	National	Screening	Healthcare and screening professionals, carers, service planners	5/6
United Kingdom (England)	Northern Cancer Alliance (NHS partnership) (39)	2019	Recommendation	Regional	Screening	Care providers, clinicians, screening practitioners, support staff	5/6
United Kingdom (England)	Leeds Teaching Hospitals NHS Trust (36)	2024	Policy (Regional)	Regional	Prevention and screening	Healthcare professionals, planners, partners in cancer services	6/6
United Kingdom (Scotland)	Scottish Government (with NHS and screening partners) (37)	2023	Policy	National	Screening	NHS Boards, professionals, planners, partners - organising and delivering screening programmes	6/6
United Kingdom (Wales)	Public Health Wales (NHS Wales) (42)	Not stated	Recommendation	Regional	Screening	Women with learning disabilities, carers, general public, support staff	5/6

Columns: organisation = publishing organisation; Type = document type; Level = implementation level; Focus = cancer control focus; Audience = target audience; AACODS = authority, accuracy, coverage, objectivity, date, Significance overall quality rating (out of 6).

### 3.3 Country distribution

**Figure 2:**
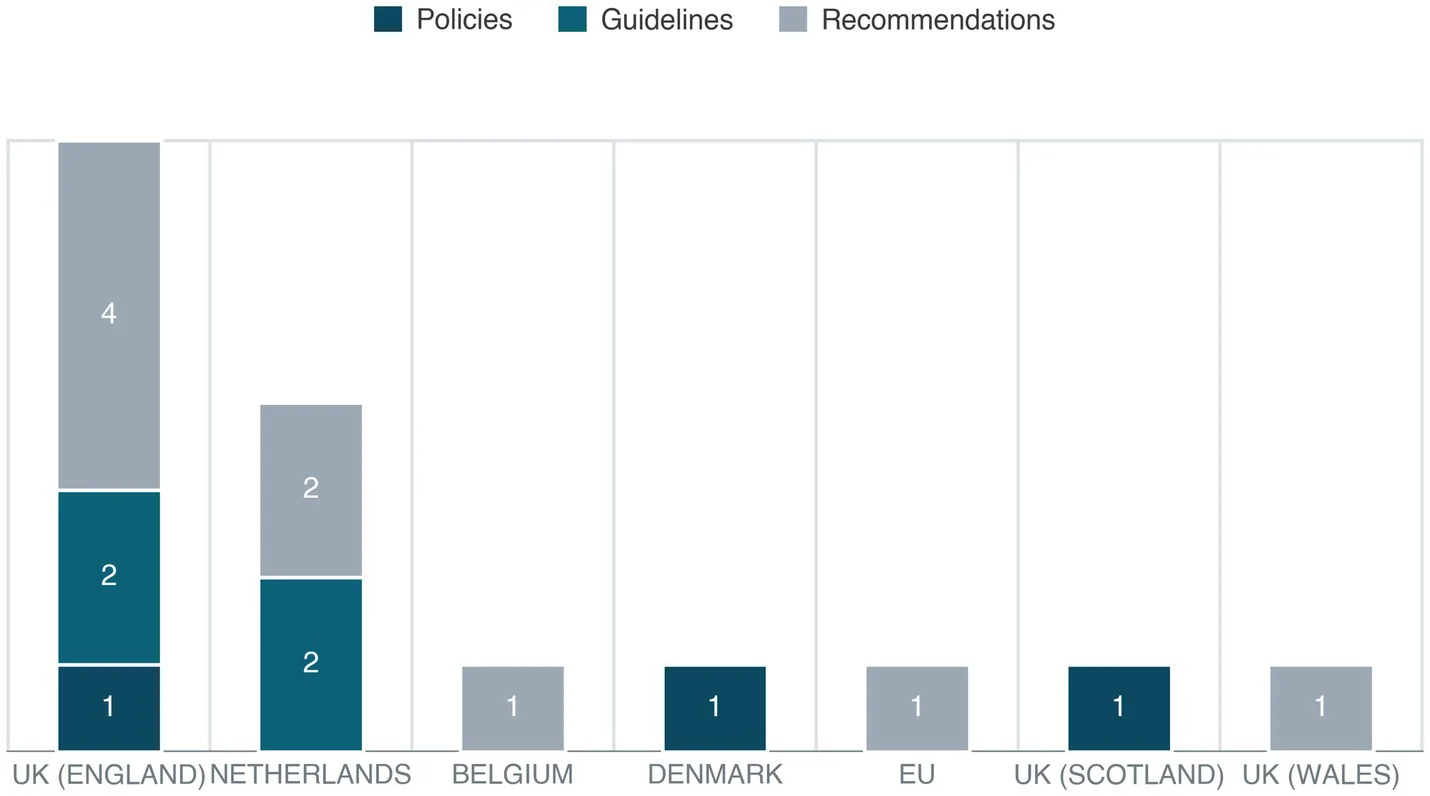
Country distribution of included cancer screening or prevention documents.

Figure 2 shows the country distribution by document type (policy, guideline, recommendation). UK and Dutch sources dominate, while the European Union, Denmark, and Belgium are each represented by a single document.

### 3.4 Cancer site and cancer control focus

**Figure 3:**
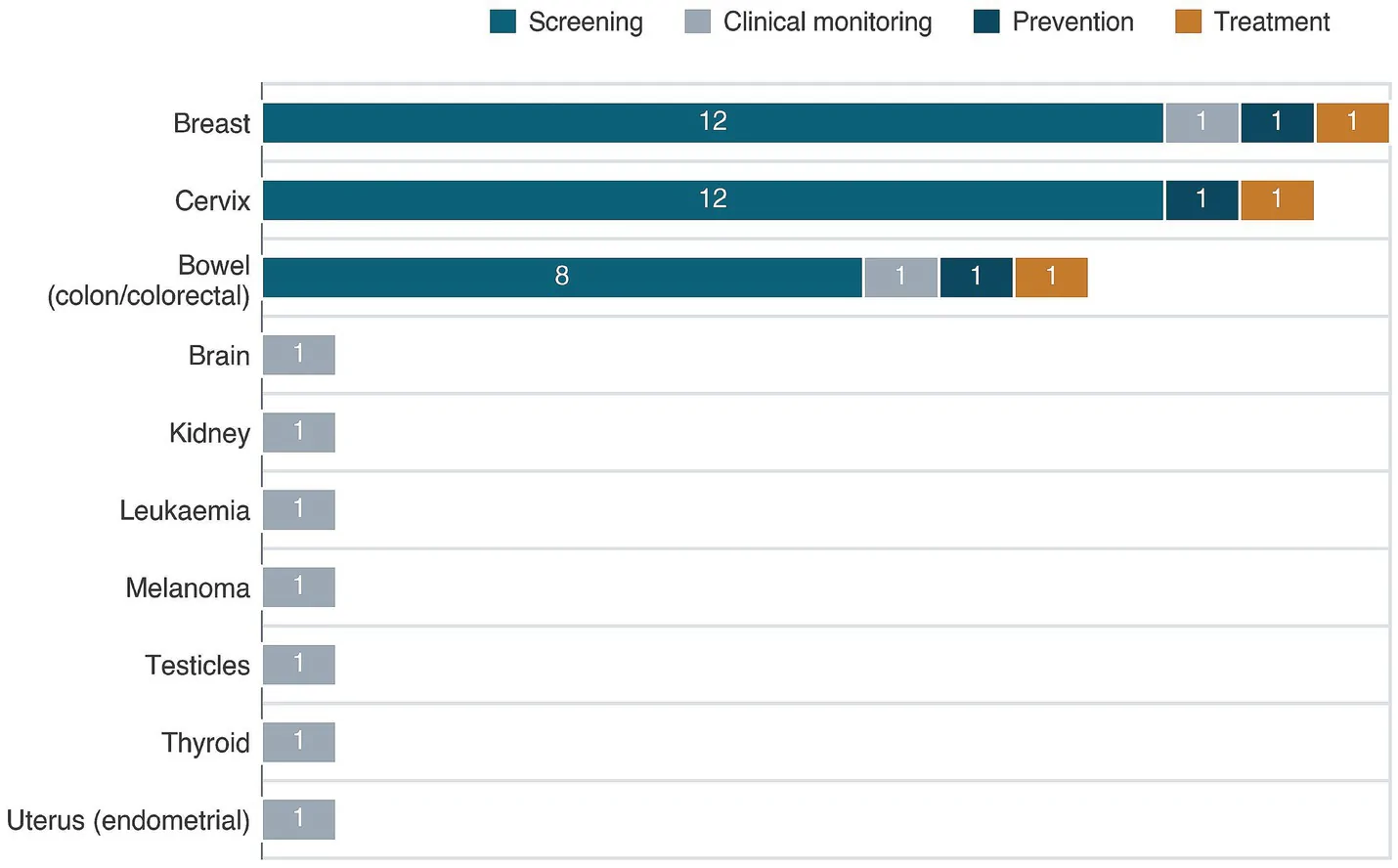
Distribution of included documents by cancer site and cancer control focus. For documents referencing multiple cancer sites, each site was counted individually.

The cancer sites addressed by the most documents were breast and cervical cancer, each the focus of 12 screening focused documents alongside one prevention focused and one treatment focused document. Breast cancer additionally appeared once under clinical monitoring, through the Cowden syndrome surveillance document, giving 15 disaggregated breast entries compared with 14 for cervical cancer. Colorectal cancer followed, with eight screening focused, one prevention focused, one treatment focused, and one clinical monitoring document (11 entries). The remaining sites each appeared in a single document under clinical monitoring only: uterine (endometrial), kidney, thyroid, melanoma, and brain cancer in the Cowden syndrome document, and leukaemia and testicular cancer in the Down syndrome document. Screening focused documents predominate, and coverage concentrates on breast, cervical, and colorectal cancer (Figure 3).

### 3.5 Synthesis

Sources included national and regional authorities (*n* = 6), local NHS structures and partnerships (*n* = 3), professional associations (*n* = 2), academic or non-governmental organisations (*n* = 2), and one patient or parent organisation, supranational body, and clinical information web resource. Some provided general system level recommendations (36, 37), whereas others gave structured pathways tailored to people with intellectual disability (38, 39); two addressed Down syndrome (40) or Cowden syndrome (41).

Most documents focused on screening. Cancer treatment is addressed in only one document (36), and clinical monitoring in two, covering Cowden syndrome (41) and Down syndrome (40). Rehabilitation and palliative care were largely absent.

Organisational adaptations to improve screening access and participation dominate. Recurring measures include Easy-Read or other adapted invitations, geographically accessible or mobile services (42), quieter or longer appointments and pre-visit familiarisation (43), invitation uptake monitoring, carer involvement, and clinical record flags identifying adults with intellectual disability (44, 45). Several documents explicitly stated that adults with intellectual disability should not be excluded from screening programmes.

Communication and consent adaptations included staff training (36, 43, 44), visual or video based materials (39, 46), collaboration with Learning Disability and Autism Teams (36, 44), and coordination between screening programmes, general practitioners, and social care services (43, 44). Additionally, consent approaches vary where some require formal capacity assessment, while others permit legal representative consent or best interests decision making within frameworks such as the Mental Capacity Act in England (46–48). This variation creates risks of both inappropriate exclusion and insufficient protection of autonomy.

Alternative breast, bowel, and cervical screening modalities included the use of general anaesthesia where procedures cannot otherwise be performed (43, 44), sedation for sigmoidoscopy or cervical screening (38, 39) and anxiolytic medication for mammography (47). Other less invasive approaches included carer presence and support, step by step explanation, relaxation techniques, environmental modification, and postponement rather than forced screening after refusal (38, 39, 43, 45, 47). Most documents treat adults with intellectual disability as a homogeneous group, except for Down syndrome (40) and Cowden syndrome (41) guidance. Supplementary Table 1 synthesises objectives and the adjustments and recommendations specific to people with intellectual disability by cancer site and cancer control focus.

### 3.6 Grouped adjustments and recommendations

#### 3.6.1 Screening related adjustments and recommendations

##### 3.6.1.1 Communication, information, and invitation strategies

Across breast, cervical, and bowel screening, the most consistent adaptation was accessible information and invitation design, including Easy-Read materials, pictorial guides, and video based explanations (39, 42, 43, 45, 46, 48, 49). Targeted invitations, pre-contact, personalised outreach, and avoidance of inappropriate removal from call-and-recall registers appeared across all three cancer sites, especially breast and cervical screening (36, 37, 43, 45, 50). One document conditionally recommended targeted rather than general communication for women with intellectual disability aged 50–69 invited for breast screening, based on low-certainty evidence (50). No included document provided recommendations specific to people with intellectual disability on smoking, alcohol reduction, diet, physical activity or HPV vaccination.

##### 3.6.1.2 Appointment adaptations and environmental adjustments

Flexible scheduling, extended appointments, and pre-visit familiarisation recurred across all three programmes (39, 42, 43, 45, 46, 49). Environmental and sensory accommodations, such as quieter appointments, familiar objects, and background music, were reported for breast and cervical screening (39, 43, 45, 46) but not bowel screening.

##### 3.6.1.3 Role of carers and support persons

Carer or support person involvement was documented across breast (39, 43, 46–49), cervical (38, 45, 46, 48, 49), and bowel screening (39, 46, 48). Roles included communication support, positioning assistance, and practical completion of home based test kits (38, 46–48). For bowel screening, where FIT or FOBt requires independent sample collection and return, multiple documents emphasised structured completion support (39, 43, 44, 46, 48).

##### 3.6.1.4 Consent, capacity, and autonomy

Consent, capacity assessment, and best interests decision making featured across breast (39, 47, 48), cervical (38, 43, 45, 46, 48), and bowel screening (39, 48). Documents emphasised informed consent with all practicable support before best interests frameworks were invoked, and that intellectual disability alone must not justify exclusion. Two documents specified that screening must not proceed after active refusal, in breast (47) and cervical screening (38, 45).

##### 3.6.1.5 Alternative screening modalities

Where standard procedures were not feasible, documents identified visual breast examination, acceptance of imperfect mammography images with planned reattempt (46, 47), CT colonography or colonoscopy under general anaesthetic (43, 44), and HPV home based self-sampling for women with disability unable to tolerate clinic based cervical examination (51). No included document provided a clear pathway for people with intellectual disability following a positive screening result, including accessible communication of results, diagnostic follow-up, referral or treatment.

##### 3.6.1.6 Health system and workforce adjustments

Systemic adaptations across all three mainstream programmes included clinical record flagging, adjusted call-and-recall processes, and liaison with specialist learning disability teams (44–46). Workforce training and awareness raising were explicit recommendations in two documents covering all three cancer sites (37, 44). Continued eligibility for cancer screening, including remaining on screening registers, was supported across breast (43, 48), cervical (43, 46, 48), and bowel screening (48). One document covering all three sites recommended no adjustments specific to intellectual disability, advising participation on the same terms as the general population (40).

##### 3.6.1.7 Prevention related recommendations

Prevention specific guidance was identified in only a single included document (36), which addressed breast, cervical, and bowel cancer collectively. The document recommended providing accessible cancer prevention information, including Easy-Read and jargon free materials, and emphasised integrating prevention activities through collaboration with specialist Learning Disability and Autism services. Coverage of this domain was therefore limited, but the recommendations were cross-cutting and applied uniformly across all three cancer sites.

##### 3.6.1.8 Clinical monitoring and surveillance

Guidance on clinical monitoring and surveillance was confined to two documents and addressed cancer sites beyond the three mainstream population screening programmes. The Cowden syndrome document (41) specified annual breast examination and breast MRI from age 25, annual thyroid ultrasound from age 12, colonoscopy every 5 years from age 35, biennial renal ultrasound from age 40, annual dermatological assessment for melanoma from age 30, and annual transvaginal ultrasound from age 30 for uterine cancer surveillance; brain tumour risk was acknowledged without a specific protocol. The Down syndrome document (40) recommended routine testicular palpation by a general practitioner or physician and prompt oncological referral if leukaemia was suspected, without endorsing routine leukaemia screening (Table 2).

**Table 2:** Grouped adjustments and recommendations specific to intellectual disability across cancer sites and the cancer control continuum.

People with intellectual disability (PWID) specific adjustment or recommendation	Breast	Cervical	Bowel/colorectal	Other cancers	Key references
Screening					
Accessible invitations & screening information (Easy-Read letters, pictorial guides, videos, clear explanations)	✓	✓	✓	–	(39, 42, 43, 45, 46, 48, 49)
Targeted or tailored invitation strategies (pre-contact, targeted messaging, avoiding inappropriate exclusion)	✓	✓	✓	–	(36, 37, 43, 45, 50)
Flexible appointment scheduling & extended time	✓	✓	✓	–	(39, 42, 43, 45, 46, 49)
Pre-visit preparation & familiarisation (meeting staff, seeing equipment beforehand)	✓	✓	✓	–	(39, 42, 43, 45, 46, 49)
Environmental and sensory adjustments (quiet times, music, familiar items)	✓	✓	–	–	(39, 43, 45, 46)
Support person/caregiver presence during screening	✓	✓	✓	–	(38, 39, 43, 45–49)
Caregiver assistance with communication, positioning, or test completion	✓	✓	✓	–	(38, 46–48)
Consent, capacity assessment & best interests decision making	✓	✓	✓	–	(38, 39, 43, 45–48)
Respect for autonomy (do not force screening if resisted)	✓	✓	–	–	(38, 45, 47)
Alternative screening approaches when standard procedures not feasible (visual breast exam, CT colonography, GA)	✓	–	✓	–	(43, 44, 46, 47)
HPV home based testing as an alternative	–	✓	—	–	(51)
Support for home based bowel screening tests (FIT/FOB)	–	–	✓	–	(39, 43, 44, 46, 48)
System flagging & administrative adjustments (LD alerts, adjusted call–recall)	✓	✓	✓	–	(44–46)
Workforce training & awareness on people with intellectual disability needs	✓	✓	✓	–	(37, 44)
Continued eligibility and inclusion in screening programmes	✓	✓	✓	–	(43, 46, 48)
Same as general population (no people with intellectual disability specific screening adjustments)	✓	✓	✓	–	(40)
Prevention					
Accessible cancer prevention information (Easy-Read, inclusive materials)	✓	✓	✓	–	(36)
Integrated prevention through collaboration with Learning Disability & Autism services	✓	✓	✓	–	(36)
Clinical monitoring/surveillance					
Enhanced surveillance due to genetic syndromes (e.g., Cowden syndrome)	✓	–	✓	Thyroid, kidney, melanoma, uterus, brain	(41)
Proactive clinical monitoring outside population screening	–	–	–	Leukaemia, testicular	(40)
Symptom awareness & caregiver monitoring when screening is unsuitable	✓	✓	✓	–	(39, 45, 47)

Across all three cancer sites covered by population screening, symptom awareness and carer supported monitoring were identified as an additional layer of clinical safety netting, particularly where formal screening was determined to be unsuitable or had been discontinued (39, 45, 47).

### 3.7 Quality of evidence

The methodological quality and credibility of all 16 included documents were appraised using the AACODS framework. Scores ranged from 4/6 to 6/6, with no document falling below this threshold. Ten documents (63%) scored 6/6 (36–38, 40, 43, 46–50), five (31%) scored 5/6 (39, 41, 42, 44, 45), and one (6%) scored 4/6 (51).

Overall the documents demonstrated authority, objectivity, accuracy, and significance although some of these judgements varied according to document type. Public Health Wales (42) lacked identifiable individual authorship, explicit references, and methodological transparency; Kinderneurologie.eu (41) and Region Hovedstaden (51) were constrained by the format of the information leaflet; NHS England (45) was similarly qualified as a recommendation document; and the NHS Cancer Screening Programmes consent guidance (48) was noted as potentially dated because it was published in 2009.

Coverage and Date accounted for most score reductions. Three documents scored “No” on Coverage (41, 44, 51), NHS England was rated “Not Applicable” on this criterion (45), and three documents scored “No” on Date (39, 42, 51). Overall, AACODS limitations were predominantly dependent on document type and do not substantially undermine the credibility of the synthesised evidence.

### 3.8 Distribution by national health expenditure

**Figure 4:**
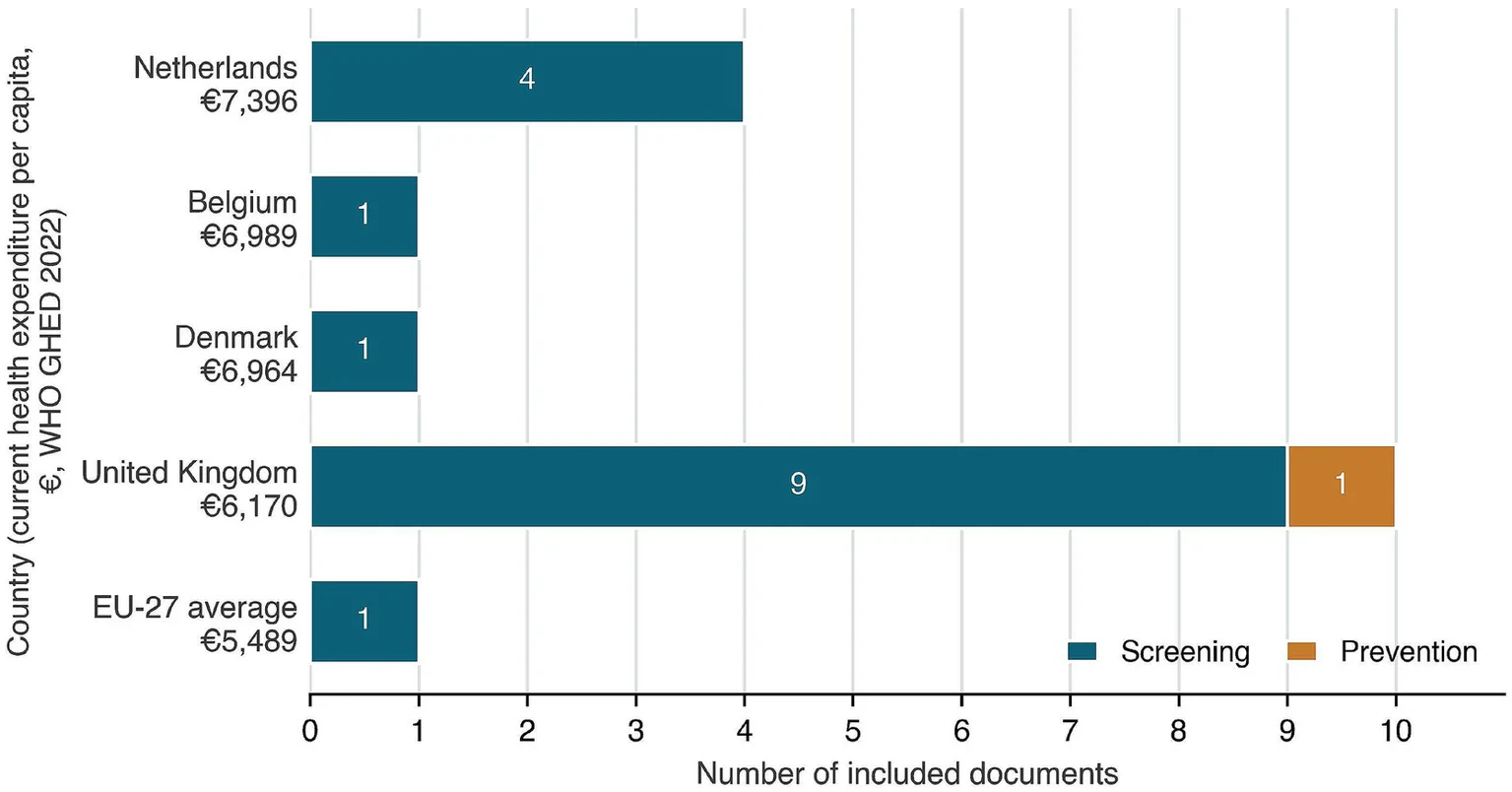
Distribution of included cancer prevention and screening documents by national health expenditure per person (euros). Values are current health expenditure per capita from the WHO Global Health Expenditure Database, 2022, expressed in purchasing power parity international dollars and converted to euros at the 2022 ECB average exchange rate (€1 = US$1.05). The single supranational document is shown at the EU-27 average, as it has no national expenditure figure. For documents addressing more than one cancer control focus, each focus was counted individually.

Resources for cancer prevention and screening identified in this review come from a small group of countries with high health expenditure. Expenditure is expressed here as current health expenditure per person, in euros, adjusted for purchasing power, from the WHO Global Health Expenditure Database (GHED), 2022. All four contributing countries spent above the EU-27 average of €5,489 per person in 2022: the Netherlands (€7,396), Belgium (€6,989), Denmark (€6,964), and the United Kingdom (€6,170) (Figure 4). The nine United Kingdom documents and the four Dutch documents account for most of the evidence, and one further document, produced by the European Commission, applies across member states and has no single national figure. The two measures rank the contributing countries differently. Their share of GDP ranged from 9.5 to 11.1%. The United Kingdom had the largest share (11.1%) but spent the least per person, while the Netherlands spent the most per person despite only a mid-range share (10.0%).

## 4 Discussion

### 4.1 Summary of principal findings

This review identified 16 policy documents from five jurisdictions (the United Kingdom, the Netherlands, Belgium, Denmark, and the European Union) addressing cancer prevention and screening for people with intellectual disability. The documents predominantly focused on screening, addressed by 15 of the 16 documents, while prevention and clinical monitoring were each represented by only one or two and the wider cancer continuum was largely absent. Most documents were written for policy makers, commissioners, and practitioners, with only three addressed to people with intellectual disability or their families, and they ranged from brief acknowledgement of additional cancer risk to detailed screening adjustment guidance. Against a background of later-stage diagnosis and substantially lower screening participation in this population (11, 22, 31, 52), the evidence points to a fragmented policy field without a consistent international approach across the screening and prevention continuum.

### 4.2 Geographic and economic distribution

Provision was concentrated in a few high expenditure systems. Nine of the 16 documents came from the United Kingdom (56%), four from the Netherlands (25%), and one each from Belgium, Denmark, and the European Union (6%). The four contributing countries all lay above the EU-27 average of €5,489 per person, yet no eligible documents were identified from lower spending countries such as Romania (€2,326) or Hungary (€2,777). None came from Germany (€8,044 per person) or Ireland (€7,871) either, despite their being among the highest per person spenders in Europe.

Expenditure alone does not explain the pattern. Established intellectual disability guidance traditions in the UK and the Netherlands (NHS consent, equity, accessible information, and workforce training frameworks, and NVAVG condition specific standards since the early 2000s), together with the legal anchoring of UK guidance in the Mental Capacity Act and reasonable adjustments duties, potentially matter more than spending. An analysis of all 27 EU member states’ National Cancer Control Programmes found that only 15% have comprehensive provisions specific to intellectual disability and only 7.5% include specialised provider training (53), and European survey evidence identifies weak disability healthcare coordination, limited policymaker awareness, and inadequate workforce training as the principal barriers (26). Higher spending on healthcare, disability, and social exclusion has been associated with smaller educational inequalities in screening uptake (54), but the German and Irish absence shows it is not sufficient.

The near-complete absence of documents from southern and eastern Europe therefore likely reflects a genuine policy gap rather than a retrieval issue, given the use of native language reviewers in each country. Concentration in higher expenditure systems does not imply adequacy within them. Substantial screening participation deficits persist despite UK and Dutch policy activity (20), and in a recent study it was also found that only about 20% of organisations across 14 countries reported a cancer prevention policy designed for this population, with most considering existing policies inadequate (26). The sole EU level document (50) suggests a role for supranational frameworks and cross-national collaboration, including COST Action networks, in extending policy beyond high expenditure systems.

### 4.3 Cancer site and continuum coverage

Of 47 disaggregated entries, 32 (68.1%) concerned screening, nine (19.1%) clinical monitoring, three (6.4%) prevention, and three (6.4%) treatment. The broader cancer control continuum (primary prevention, treatment, survivorship, and palliative care) is virtually unaddressed for people with intellectual disability.

#### 4.3.1 Screening as the near-exclusive policy focus

The included documents concentrated on screening, developing adjustments specific to intellectual disability within mainstream programmes. This reflects the structure of European cancer control, where organised population-based breast, cervical, and colorectal screening has been the primary vehicle for investment (55), with adjustments embedded in call-and-recall systems, consent processes, and screening pathways. The focus answers a documented need. Participation deficits of approximately 20 percentage points across breast, cervical, and colorectal screening have been recorded in the Netherlands (20), and a systematic review identifies structural, communicative, and attitudinal barriers as the principal drivers of low uptake (24). The documents respond predominantly to these access level barriers.

#### 4.3.2 Limits of the access only frame

The included documents addressed screening participation but offered no dedicated symptom reporting or self-examination guidance for adults with intellectual disability. This gap matters because equitable screening access does not by itself resolve cancer outcome disparities. Adults with intellectual disability present later for breast and colorectal cancer (14), a substantial proportion of their colorectal cancer deaths in England occur below the screening age threshold (13), and diagnostic overshadowing (clinical signs misattributed to a person’s intellectual disability rather than investigated as possible cancer) operates across the diagnostic and treatment pathway (17). Because organised European screening covers only breast, cervical, and colorectal cancer, earlier detection elsewhere depends on symptom recognition rather than a screening programme, yet the documents addressed this only as a carer supported safety net where screening was unsuitable (39, 45, 47).

#### 4.3.3 Continuum gaps beyond screening

No specific guidance was identified on smoking, alcohol reduction, diet, physical activity or HPV vaccination. Guidance on managing positive screening results was also absent. Only one document addressed prevention (36), without recommendations on lifestyle risk modification, vaccination, or health promotion specific to intellectual disability, despite disproportionate exposure to modifiable cancer risk factors (11). The European Code Against Cancer reports that a substantial proportion of cancers are preventable through modifiable risk factors, yet how its recommendations translate into provision for people with intellectual disability remains underdeveloped (18). Treatment accounted for three disaggregated entries (6.4%), all from one source (36), while survivorship and palliative care received no dedicated policy representation. This contrasts with evidence of sparse survivorship research, less intensive treatment, and poorer survival among adults with intellectual disability (16, 31, 56). The nine clinical monitoring entries all came from the Netherlands and addressed syndrome specific protocols for Cowden syndrome and Down syndrome rather than a population level surveillance approach. This points to a need for cross-national knowledge transfer and comparable syndrome specific guidance.

Taken together, this suggests a policy field that has prioritised the most established and institutionally embedded domain of cancer control (screening access) but has yet to extend its scope across the full cancer continuum.

### 4.4 Adjustments specific to intellectual disability: coverage and gaps

The adjustments documented across the included guidance were notably consistent. Their stated objectives centred on reducing inequalities, improving screening access, and supporting informed, person-centred care, while the practices they specified clustered on a recurring set: accessible information, communication support, carer or supporter involvement, and reasonable adjustments to standard pathways. This consistency aligns with evidence that tailored interventions improve healthcare access and engagement for adults with intellectual disability more broadly (30), who experience lower screening participation and poorer outcomes (15, 52) driven by individual and system level barriers (23).

Despite this most documents lacked depth. For example, objectives were generally broad and strategic with limited emphasis on implementation, a pattern also seen in wider cancer policy research (32). While adjustment level guidance was more operational, its level of detail varied and it was rarely accompanied by implementation or evaluation evidence. Both objectives and adjustments were weighted toward screening and early detection, with treatment, survivorship, and palliative care underrepresented. The recurrence of the same adjustments across otherwise heterogeneous documents nonetheless indicates that they are concrete and consistent enough to function as standard components of equitable screening approaches rather than *ad hoc* accommodations.

### 4.5 The policy–practice gap

Beyond its geographic concentration, the evidence base itself is fragmented and low in visibility. None of the included guidance came from peer-reviewed literature. No eligible documents were retrieved from the five academic databases searched, despite a combined deduplicated total of 2,394 records, and all 16 included documents were retrieved from grey literature searches. This guidance is therefore disseminated outside peer-reviewed channels, with limited visibility to the clinicians, researchers, and policymakers who may rely on academic databases. Stronger integration of policy development, academic publication, and accessible grey literature repositories would help close this gap.

The documents were also heterogeneous and varied in type and scope. They ranged from supranational recommendations and national policies to regional toolkits and public facing leaflets. Within the United Kingdom, national, devolved, and regional guidance coexisted without a unifying implementation framework, while the sole supranational document (50) addressed one aspect of one screening programme on low-certainty evidence. Several documents were more than a decade old (38, 43, 47, 48), which raises questions about its continued relevance as consent law, mental capacity practice, screening technologies and clinical protocols evolve. Most European jurisdictions therefore need to develop, formalise, disseminate, periodically update, and extend guidance specific to intellectual disability beyond screening across the full cancer continuum.

### 4.6 Strengths and limitations

This review provides a comprehensive overview of European policy frameworks, guidelines, and recommendations for cancer prevention and screening for people with intellectual disability. Through quantifying and categorising existing evidence, the study presents a structured approach to identifying gaps in policy provision for this population. The inclusion of multidisciplinary and multi-professional reviewers across countries of differing average incomes supports a detailed understanding of cross-European variation. The use of native language reviewers per country, each with policy domain familiarity, supports the interpretation that documented absence reflects genuine policy absence rather than retrieval failure.

A procedural limitation concerns loss of the deduplication log during searching after a remote file management error. The exact number of duplicate records removed before screening could not be reported, and only the post-deduplication screened total (*n* = 2,394) could be verified. This does not affect the final included document set or synthesis, but it departs from full PRISMA 2020 transparency and constrains reproducibility of the pre-screening phase.

A further limitation is the geographic concentration of included documents, with nine of 16 (56%) originating from the United Kingdom. The synthesised recommendations predominantly reflect a single health system’s legislative framework, notably the Mental Capacity Act, and may not be directly transferable to countries operating under different legal and organisational structures. The near absence of guidance from most EU member states is itself a substantive finding but constrains generalisability across Europe.

Finally, although searches used native language reviewers per country, document availability may have varied, and non-publicly accessible materials may have been missed. The study also relies on document analysis and does not assess implementation or effectiveness.

### 4.7 Implications for policy and practice

The synthesis carries direct implications for clinical practice and service design. Reasonable adjustments are a systematic requirement for equitable care and should not be treated as optional enhancements. Across breast, cervical, and bowel screening, accessible invitations, extended appointments, pre-visit familiarisation, environmental modifications, carer support, and adapted consent processes show that standard pathways often do not fit communication, understanding, sensory, and support needs. Several documents state that these adjustments should be anticipated and provided rather than left for individuals or carers to request (39, 43).

Where standard pathways are not feasible, documented alternatives include visual breast examination, acceptance of technically imperfect mammography images, carer involvement in positioning and explanation (46–48), individual cervical screening capacity assessment without exclusion based on assumed sexual inactivity or procedural intolerance (38, 43, 45, 46, 48), HPV home based self-sampling where clinic based examination cannot be tolerated (51), carer support for FIT/FOBt sample collection and return (39, 43, 44, 46, 48), and CT colonography or colonoscopy under general anaesthetic when bowel screening pathways cannot be completed (25, 43, 44).

Capacity should be assessed individually, procedure specifically, and time specifically, with flexible scheduling where capacity fluctuates (43, 45, 46, 48). Familiarity with supported and best interest decision making should be expected of clinicians outside specialist learning disability services, an unmet training need identified in several documents (37, 44). Intellectual disability alone never justifies removal from a screening register or withholding an invitation (43, 46, 48). Providing these adjustments requires adequate support systems, including trained personnel which should be explicitly resourced rather than relying on individual professionals, families or support staff.

Carers are central to accessible information, appointment accompaniment, home based test completion, and symptom monitoring, but should not be treated as an assumed informal resource. The supranational document (50) notes that targeted invitations may require additional carer time and effort, which implementation planning should consider. For people with Cowden syndrome and Down syndrome, the surveillance expectations (40, 41) also affect annual health checks and primary care specialist referral pathways. The adjustments documented here represent a minimum standard of equitable care, not a ceiling of ambition.

### 4.8 Recommendations

Five headline recommendations are presented below. A further seven policy considerations are provided in Supplementary Material (Additional policy considerations). Each recommendation uses *should* for primary actions and *may* for secondary actions.

- *Recommendation 1.* The European Commission should establish a coordinated, EU wide policy framework for inclusive cancer screening that explicitly addresses the needs of people with intellectual disability.

Rationale: The European Commission Initiative on Breast Cancer guidance (50) addressed one aspect of one programme on low-certainty, conditional evidence; this is not an EU policy framework and leaves the geographic inequity documented in this review unaddressed.

- *Recommendation 2.* The European Commission Initiative on Breast Cancer should be extended to cover cervical and bowel cancer screening, with provisions specific to intellectual disability across all three mainstream programmes.

Rationale: The ECIBC evidence-to decision framework shows that structured EU level recommendations on adaptations specific to intellectual disability are feasible (50); extending this model to cervical and bowel screening would close the supranational guidance gap documented here.

- *Recommendation 3.* Member states without existing cancer screening guidance specific to intellectual disability should treat its development as an urgent health equity obligation.

Rationale: Only Belgium, Denmark, and the Netherlands among EU member states contributed eligible documents. All other EU member states lacked eligible guidance. UK guidance shows that structured, actionable screening guidance specific to intellectual disability is feasible within a public health system. Because population screening operates across the EU, this is a remediable health inequality requiring guidance adapted to national legislation, health system structures, and languages.

- *Recommendation 4.* Member states should enact or strengthen legislative frameworks that mandate reasonable adjustments in cancer screening for people with intellectual disability.

Rationale: Operational recommendations on consent, capacity assessment, best interests decision making, and screening register maintenance are anchored in national law, notably the UK Mental Capacity Act. Equivalent grounding may be needed to make guidance enforceable, so governments should review and strengthen disability rights and healthcare legislation where necessary.

- *Recommendation 5.* National cancer screening programme commissioners should embed adjustments specific to intellectual disability as standard operational components of breast, cervical, and bowel screening programmes.

Rationale: Documented adjustments (accessible invitations, extended and flexible appointments, pre-visit familiarisation, environmental modifications, carer involvement, and adapted consent processes) are consistent enough to be standard operational elements rather than reactive accommodations. Commissioners should audit practice, identify gaps, and resource change.

### 4.9 Future research

Although this review did not have an explicit empirical research focus, several research implications follow from the findings. In jurisdictions where cancer prevention and screening policies specific to intellectual disability exist, evaluation of their impact would be valuable: on policy awareness, particularly among clinicians; on behaviours such as preventive action and screening uptake; and on outcomes such as cancer detection rates and stage at diagnosis, particularly given the limited evidence on the benefits and harms of cancer screening for adults with intellectual disability (57). These measures would indicate whether current policies are reducing late-stage diagnoses in this population. A complementary review of policies specific to people with intellectual disability across other areas of cancer care, including treatment and palliative care, is also warranted, and would likely have a more localised focus. Sustaining this work will depend on continued cross-national collaboration. A funded knowledge network linking researchers, policymakers, and disability and cancer organisations across Europe would allow comparative work and coordinated evaluation of new guidance to continue beyond the lifetime of any single project.

## 5 Conclusion

European cancer prevention and screening policy provision for people with intellectual disability is uneven, concentrated in a small group of higher expenditure healthcare systems, and predominantly oriented toward access to organised screening. Sixteen documents from five jurisdictions (the United Kingdom, the Netherlands, Belgium, Denmark, and the European Union) were identified. No eligible guidance was retrieved from the majority of European member states. Where guidance exists, it concentrates on breast, cervical, and bowel cancer screening, with prevention, treatment, and survivorship largely unaddressed. The adjustments documented are consistent and operationally specific, but variation in document type, implementation level, and currency suggests fragmentation rather than coordination. Closing the policy–practice gap will require national commissioning of inclusive guidance where it is absent, extension of existing guidance across the full cancer continuum, and EU level coordination to support cross-national knowledge transfer. Future policy should also address modifiable cancer risk factors, including HPV vaccination, management following positive screening results, and the workforce capacity required to provide reasonable adjustments throughout the cancer pathway. The COST CUPID Action provides one infrastructure through which such coordination could be advanced.

## Footnotes

1
https://osf.io/f5zqk/
