# Supplementary material for: Cancer prevention and screening for people with intellectual disabilities in Europe: a systematic review and recommendations for policy and practice
Source: Front Public Health. 2026 Aug 20;14:1925665. doi: 10.3389/fpubh.2026.1925665 (PMC13539603; doi:10.3389/fpubh.2026.1925665)
Supplement: Supplementary file 1 [file Table_1.docx]

Supplementary Material

Supplementary material for: A Systematic Review of European Policy Frameworks, Guidelines and Recommendations for Cancer Prevention and Screening in People with Intellectual Disabilities.

# Search strategy

This file documents the search strategy for the review. It sets out the search framework, the information sources searched, a complete worked example of a database search string, and the records retrieved at each source. Per country search strings are held by the country teams and are available on request.

## Search framework

The strategy used the Population, Concept, Context framework adapted for policy and grey literature review, with an added Country component for cross-national consistency. Searches covered the 53 member states of the World Health Organisation European Region and were run to August 2025.

| Element | Definition used in this review |
| --- | --- |
| Population | People with intellectual disabilities, including those described in source documents as having learning disabilities or developmental disabilities, and conditions within the intellectual disability umbrella. No age restrictions were applied. |
| Concept | Cancer of any type or location. Prevention and screening terms were not used as search filters, to avoid excluding documents of broader scope. |
| Context | Publicly available documents intended to inform policy or practice, including policies, guidelines, legislation, recommendations, information leaflets, and strategic reports. |
| Country | A single country of interest, searched with country specific terms, including translation into the national language(s) where required. |

### Population search terms

Uniform country search terms covered the intellectual disability umbrella: intellectual disability, learning disability, developmental disability, Down syndrome, Fragile X syndrome, Williams syndrome, DiGeorge syndrome, 22q11 deletion syndrome, Prader–Willi syndrome, Angelman syndrome, Wolf–Hirschhorn syndrome, Cri du Chat syndrome, xeroderma pigmentosum, Fanconi anaemia, Nijmegen breakage syndrome, neurofibromatosis type 1, Bloom syndrome, Rubinstein–Taybi syndrome, Klippel–Trenaunay syndrome, Saethre–Chotzen syndrome, Cowden syndrome, Costello syndrome, Noonan syndrome, and disorders of sex development.

## Information sources

| Source type | Sources searched |
| --- | --- |
| Academic databases | PubMed/MEDLINE, Embase, Web of Science, PsycINFO, CINAHL |
| Grey literature portals | Google Scholar, Policy Commons |
| Structured web search | Google, restricted to the first 100 results per country per search date |
| Snowball searching | National databases and relevant organisations responsible for cancer prevention or screening policy |

One reviewer searched and screened each country, and a second reviewer independently verified eligibility, with discrepancies resolved by consensus. Native language reviewers searched each country, with translation where required. Per country logs recorded reviewer identifiers, search dates, stage counts, the first 100 Google URLs, evidence of structured searches, and the included documents.

## Worked example — complete search string (United Kingdom)

The full United Kingdom database search string is given below as an illustrative example. It was reviewed by an academic librarian. Boolean operators, parentheses, and truncation were used to balance sensitivity and specificity. Per country strings adapted the same four concept blocks with country specific terms and are available on request.

("learning disabil*" OR "intellectual disabil*" OR "developmental disabil*")
AND
(cancer OR oncology OR carcinoma OR malign* OR tumor OR neoplasm*)
AND
(policy OR policies OR guidance* OR law* OR legal* OR guidelin* OR strateg* OR legislat* OR statutory OR governan* OR regulation* OR "health act*")
AND
("United Kingdom" OR "gov.uk" OR "nhs.uk" OR "nice.org.uk" OR "parliament.uk")

| Concept block | Terms |
| --- | --- |
| Population | “learning disabil*” OR ”intellectual disabil*” OR “developmental disabil*” |
| Cancer | cancer OR oncology OR carcinoma OR malign* OR tumor OR neoplasm* |
| Policy | policy OR policies OR guidance* OR law* OR legal* OR guidelin* OR strateg* OR legislat* OR statutory OR governan* OR regulation* OR “health act*” |
| Country | “United Kingdom” OR “gov.uk” OR “nhs.uk” OR “nice.org.uk” OR “parliament.uk” |

## Records retrieved by source

Record counts at each stage of identification, screening, and inclusion are shown in the PRISMA 2020 flow diagram (Figure 1). The per source counts below summarise identification.

| Source | Records |
| --- | --- |
| PubMed/MEDLINE | 1,368 |
| Embase | 867 |
| Web of Science | 129 |
| PsycINFO | 20 |
| CINAHL | 10 |
| Academic databases, after deduplication | 2,394 |
| Google | 1,737 |
| Google Scholar | 3,490 |
| Targeted organisational portals | 985 |
| European Commission | 4 |
| Policy Commons | 663 |
| Grey literature records (total) | 6,879 |

All database sourced records were excluded at title and abstract screening; the included evidence base derives entirely from grey literature sources. Of the records identified through grey literature methods, 141 reports were sought and retrieved in full text, and 16 documents met the inclusion criteria.

Because Google returns large and non-reproducible hit counts, screening was restricted to the first 100 results per country per search date, so the Google counts reflect records screened rather than total returned hits. Duplicate removal used database native functions for indexed sources and manual country pair review for grey literature records. The consolidated deduplication log was lost through a remote file management error, so only the post-deduplication screened total of 2,394 could be verified.

# PRISMA 2020 checklist

Locations are given by manuscript section. Page numbers will be inserted once the manuscript is paginated for submission.

This review synthesised policy frameworks, guidelines, recommendations, information leaflets, and legislative documents rather than primary intervention studies. Three reporting consequences follow, and are noted in the checklist below. First, methodological quality and credibility were appraised with the Authority, Accuracy, Coverage, Objectivity, Date, Significance (AACODS) checklist in place of a study risk-of-bias tool. Second, synthesis was narrative, so the items specific to meta-analysis, statistical heterogeneity, sensitivity analysis, and quantitative effect measures are not applicable. Third, certainty-of-evidence grading (for example GRADE) was not applied, as the corpus comprises policy and grey literature documents rather than outcome data.

| Section and Topic | Item # | Checklist item | Location where item is reported |
| --- | --- | --- | --- |
| **TITLE** |  |  |  |
| Title | 1 | Identify the report as a systematic review. | Title |
| **ABSTRACT** |  |  |  |
| Abstract | 2 | See the PRISMA 2020 for Abstracts checklist. | Abstract |
| **INTRODUCTION** |  |  |  |
| Rationale | 3 | Describe the rationale for the review in the context of existing knowledge. | Introduction; Introduction — Rationale |
| Objectives | 4 | Provide an explicit statement of the objective(s) or question(s) the review addresses. | Introduction — Objective |
| **METHODS** |  |  |  |
| Eligibility criteria | 5 | Specify the inclusion and exclusion criteria for the review and how studies were grouped for the syntheses. | Methods — Eligibility criteria |
| Information sources | 6 | Specify all databases, registers, websites, organisations, reference lists and other sources searched or consulted to identify studies. Specify the date when each source was last searched or consulted. | Methods — Data sources and search strategy (searches run to August 2025); Supplementary Methods — Search strategy |
| Search strategy | 7 | Present the full search strategies for all databases, registers and websites, including any filters and limits used. | Methods — Data sources and search strategy; Supplementary Methods — Search strategy (complete UK search string; per country strings available on request) |
| Selection process | 8 | Specify the methods used to decide whether a study met the inclusion criteria of the review, including how many reviewers screened each record and each report retrieved, whether they worked independently, and if applicable, details of automation tools used in the process. | Methods — Study selection and data extraction (one reviewer per country, second independent verification, consensus); Methods — Use of an AI tool |
| Data collection process | 9 | Specify the methods used to collect data from reports, including how many reviewers collected data from each report, whether they worked independently, any processes for obtaining or confirming data from study investigators, and if applicable, details of automation tools used in the process. | Methods — Study selection and data extraction (standardised Excel template; subset or full independent checking by the second country reviewer); Methods — Use of an AI tool |
| Data items | 10a | List and define all outcomes for which data were sought. Specify whether all results that were compatible with each outcome domain in each study were sought (e.g. for all measures, time points, analyses), and if not, the methods used to decide which results to collect. | Methods — Study selection and data extraction (primary outcome: identification and characterisation of documents addressing cancer prevention or screening for people with intellectual disabilities) |
|  | 10b | List and define all other variables for which data were sought (e.g. participant and intervention characteristics, funding sources). Describe any assumptions made about any missing or unclear information. | Methods — Study selection and data extraction (document title, organisation, country and scope, publication date, cancer type, policy focus, aim, target audience, intellectual disability relevant content) |
| Study risk of bias assessment | 11 | Specify the methods used to assess risk of bias in the included studies, including details of the tool(s) used, how many reviewers assessed each study and whether they worked independently, and if applicable, details of automation tools used in the process. | Methods — Quality appraisal (AACODS checklist used in place of a study risk-of-bias tool, as appropriate to policy and grey literature documents; two reviewers per document, consensus) |
| Effect measures | 12 | Specify for each outcome the effect measure(s) (e.g. risk ratio, mean difference) used in the synthesis or presentation of results. | Not applicable — narrative synthesis of policy documents; no quantitative effect measures |
| Synthesis methods | 13a | Describe the processes used to decide which studies were eligible for each synthesis (e.g. tabulating the study intervention characteristics and comparing against the planned groups for each synthesis (item #5)). | Methods — Synthesis (documents grouped by cancer site, cancer control focus, and geographical/administrative level) |
|  | 13b | Describe any methods required to prepare the data for presentation or synthesis, such as handling of missing summary statistics, or data conversions. | Methods — Synthesis (documents addressing multiple cancer sites disaggregated into site specific components) |
|  | 13c | Describe any methods used to tabulate or visually display results of individual studies and syntheses. | Methods — Synthesis; Results (Tables 1–2; Figures 1–4; Supplementary Tables 1–2) |
|  | 13d | Describe any methods used to synthesize results and provide a rationale for the choice(s). If meta-analysis was performed, describe the model(s), method(s) to identify the presence and extent of statistical heterogeneity, and software package(s) used. | Methods — Synthesis (narrative synthesis; meta-analysis not appropriate given heterogeneity of document type, scope, and reporting format) |
|  | 13e | Describe any methods used to explore possible causes of heterogeneity among study results (e.g. subgroup analysis, meta-regression). | Not applicable — narrative synthesis |
|  | 13f | Describe any sensitivity analyses conducted to assess robustness of the synthesized results. | Not applicable — narrative synthesis |
| Reporting bias assessment | 14 | Describe any methods used to assess risk of bias due to missing results in a synthesis (arising from reporting biases). | Not applicable — no quantitative synthesis; assessment of reporting bias due to missing results not conducted |
| Certainty assessment | 15 | Describe any methods used to assess certainty (or confidence) in the body of evidence for an outcome. | Not applicable — certainty grading (e.g. GRADE) not used; document credibility appraised with AACODS (Methods — Quality appraisal) |
| **RESULTS** |  |  |  |
| Study selection | 16a | Describe the results of the search and selection process, from the number of records identified in the search to the number of studies included in the review, ideally using a flow diagram. | Results — Search results; Figure 1 (PRISMA 2020 flow diagram) |
|  | 16b | Cite studies that might appear to meet the inclusion criteria, but which were excluded, and explain why they were excluded. | Results — Search results (full-text exclusions with reasons: not specific to cancer 27, not specific to people with intellectual disability 8, general mention only 4, other 86) |
| Study characteristics | 17 | Cite each included study and present its characteristics. | Results — Characteristics of included documents; Table 1 |
| Risk of bias in studies | 18 | Present assessments of risk of bias for each included study. | Results — Quality of evidence (AACODS); Supplementary Table 2 (per document AACODS appraisal) |
| Results of individual studies | 19 | For all outcomes, present, for each study: (a) summary statistics for each group (where appropriate) and (b) an effect estimate and its precision (e.g. confidence/credible interval), ideally using structured tables or plots. | Not applicable — no quantitative outcomes; per document objectives and adjustments presented in Supplementary Table 1 and Table 2 |
| Results of syntheses | 20a | For each synthesis, briefly summarise the characteristics and risk of bias among contributing studies. | Results — Synthesis; Results — Quality of evidence (AACODS) |
|  | 20b | Present results of all statistical syntheses conducted. If meta-analysis was done, present for each the summary estimate and its precision (e.g. confidence/credible interval) and measures of statistical heterogeneity. If comparing groups, describe the direction of the effect. | Not applicable — narrative synthesis; no meta-analysis |
|  | 20c | Present results of all investigations of possible causes of heterogeneity among study results. | Not applicable — narrative synthesis |
|  | 20d | Present results of all sensitivity analyses conducted to assess the robustness of the synthesized results. | Not applicable — narrative synthesis |
| Reporting biases | 21 | Present assessments of risk of bias due to missing results (arising from reporting biases) for each synthesis assessed. | Not applicable — no quantitative synthesis |
| Certainty of evidence | 22 | Present assessments of certainty (or confidence) in the body of evidence for each outcome assessed. | Not applicable — certainty grading not used; AACODS appraisal in Results — Quality of evidence |
| **DISCUSSION** |  |  |  |
| Discussion | 23a | Provide a general interpretation of the results in the context of other evidence. | Discussion — interpretation |
|  | 23b | Discuss any limitations of the evidence included in the review. | Discussion — Strengths and limitations |
|  | 23c | Discuss any limitations of the review processes used. | Discussion — Strengths and limitations (deduplication log loss; Google first-100 restriction; geographic concentration; language coverage) |
|  | 23d | Discuss implications of the results for practice, policy, and future research. | Discussion — recommendations; Implications for practice; Future research |
| **OTHER INFORMATION** |  |  |  |
| Registration and protocol | 24a | Provide registration information for the review, including register name and registration number, or state that the review was not registered. | Methods — Protocol registration and ethics (PROSPERO, registration number CRD420251140644) |
|  | 24b | Indicate where the review protocol can be accessed, or state that a protocol was not prepared. | Methods — Protocol registration and ethics (PROSPERO record: https://www.crd.york.ac.uk/prospero/display_record.php?ID=CRD420251140644; project materials on the Open Science Framework, https://osf.io/f5zqk/) |
|  | 24c | Describe and explain any amendments to information provided at registration or in the protocol. | Methods — Protocol registration and ethics; any amendments are recorded on the PROSPERO registration (CRD420251140644) |
| Support | 25 | Describe sources of financial or non-financial support for the review, and the role of the funders or sponsors in the review. | Funding |
| Competing interests | 26 | Declare any competing interests of review authors. | Competing interests |
| Availability of data, code and other materials | 27 | Report which of the following are publicly available and where they can be found: template data collection forms; data extracted from included studies; data used for all analyses; analytic code; any other materials used in the review. | All included documents were publicly available at the time of extraction (Methods — Protocol registration and ethics); per country search logs and the standardised data extraction template are available on request (Supplementary Methods — Search strategy) |

*From:* Page MJ, McKenzie JE, Bossuyt PM, Boutron I, Hoffmann TC, Mulrow CD, et al. The PRISMA 2020 statement: an updated guideline for reporting systematic reviews. BMJ 2021;372:n71. doi: 10.1136/bmj.n71.

# Use of an AI tool

ChatGPT (OpenAI) was used by one author as a supplementary reliability cross-check on both the searches and the data extraction, after the human led work was complete.

## Tool and run details

| Field | Value |
| --- | --- |
| Tool | ChatGPT (OpenAI) |
| Model / version | GPT-4o (OpenAI); exact version not recorded at the time of use |
| Search mode (Prompt 1) | Standard web search (live browsing) |
| Date(s) of use | June to August 2025 |
| Yield | No additional eligible documents identified |

## Prompt framework

The completeness search (Prompt 1) used a plain-language instruction. The data-extraction cross-check (Prompt 2) used a structured **Role–Context–Task–Instructions–Constraints–Output** prompt, with each element wrapped in XML tags as delimiters so the model could separate fixed instructions from inputs.

## Prompt 1 — Supplementary search (completeness cross-check)

Purpose: for countries where the human-led search returned no eligible documents, one member of the review team used ChatGPT with live web search to confirm that no eligible national documents had been missed. This was a live online search of the open web, not a search over a supplied document set or an abstract database. No additional eligible documents were identified.

The instruction given to the tool was a plain-language request. A representative form, with the country name substituted per run, is:

Search for official national documents from {COUNTRY} related to cancer
prevention and/or cancer screening for people with intellectual disabilities.
Include policies, national strategies, clinical guidelines, recommendations,
health service guidance, and official reports published by government bodies,
ministries of health, national health services, public health agencies, or
recognised professional organisations.

## Prompt 2 — Data extraction quality assurance (reliability cross-check)

<role>
You are a meticulous data extraction reviewer for a structured policy review. You
independently extract structured data from the document attached to this conversation and
quote the text that supports each field.
</role>

<context>
The document is attached to this conversation. A country reviewer pair has already extracted structured data from it. Your
independent extraction will be compared, outside this conversation, against theirs as a
reliability cross-check. You are not given the human extraction and must not guess it.
Extract using the schema in <schema>.
</context>

<schema>
- objective(s): what the document recommends or mandates on cancer prevention/screening.
- pwid_adjustments: any adaptation specific to adults with intellectual disabilities
 (e.g. accessible communication, consent processes, caregiver involvement, system level
 adjustments). Record "none stated" if absent.
- cancer_site(s): breast, cervical, colorectal, prostate, ovarian, or other (name it).
- cancer_control_focus: screening, prevention, treatment, or clinical monitoring.
- level: national, regional, or supranational.
- document_type: policy, guideline, recommendation, leaflet, or legislation.
</schema>

<inputs>
 <attached_document>
 The source document is attached to this conversation. Read it in full and extract only
 from this attached document.
 </attached_document>
</inputs>

<task>
1. Independently extract each field in <schema> using ONLY the attached document.
2. For every field, quote the exact supporting sentence(s) from the document, or write
 "no supporting text found".
3. Do not compare against, infer, or speculate about any other extraction.
</task>

<instructions>
- Extract only what the attached document states. Do not use outside knowledge or infer
 beyond the text.
- Read the whole document before extracting.
- Where a field is not addressed, record "none stated" (for pwid_adjustments) or "not
 specified", rather than guessing.
- If the attachment is unreadable or incomplete, say so rather than proceeding.
</instructions>

<constraints>
- No fabrication: every field value must be backed by a quoted span from the attached
 document.
- Use person-first terminology.
- Keep quotes verbatim and short (25 words or fewer each).
</constraints>

<output_format>
Return one row per schema field, and nothing else:

| Field | Extracted value | Supporting quote (verbatim) |

If a field cannot be extracted, write the reason in the supporting quote cell.
</output_format>

# Supplementary Table 1

**Supplementary Table 1.** Objectives and intellectual disability specific adjustments and recommendations of the 16 included documents, stratified by cancer site and cancer control focus. Rows are grouped under bold cancer site sub-headers. A document recurring across several sites or control foci appears once per applicable row. “#” in the source denotes the terminology footnote (UK/NHS “learning disability” usage; see the §3.6 footnote of the main report). Based on Vladimir Vukovic’s 1 June 2026 results document. The cancer control focus of the Cowden syndrome document (ref 7) has been corrected from screening to clinical monitoring for its breast, colorectum, and uterine (endometrial) rows. The source document specifies hereditary high-risk surveillance (annual breast MRI from age 25, five-yearly colonoscopy from age 35, annual transvaginal ultrasound from age 30), not population screening. This aligns the table with Figure 3, in which colorectum is grouped within bowel (colon/colorectal).

| Ref | Organisation | Country | Cancer site / control focus | Objectives | Adjustments and recommendations for adults with intellectual disability |
| --- | --- | --- | --- | --- | --- |
| 14 | Leeds Teaching Hospitals NHS Trust | United Kingdom (England) | Bowel (colon / colorectal)⏎Prevention | 1. Set a strategic framework – Guide cancer services across prevention, treatment, survivorship, and end-of-life care. 2. Address population needs and inequalities – Integrate prevention and early detection to reduce disparities. 3. Promote quality and person -centred care – Ensure equitable, high-quality care aligned with The Leeds Way values. 4. Support innovation and research – Translate research into improved high quality patient centered diagnostics, treatment, and care pathways. 5. Plan service improvements – Strengthen cancer pathway capabilities through partnership and continuous improvement. | The strategy does not list site specific recommendations for breast, cervical, or bowel cancer, but a general recommendation, as follows: Work with the Learning Disabilities and Autism Team to make cancer prevention information equitable and accessible. |
| 8 | NHS Cancer Screening Programmes | United Kingdom (England) | Bowel (colon / colorectal)⏎Screening | 1. Clarify informed consent – Ensure participation is voluntary and based on understanding screening benefits, risks, and limitations. 2. Guide consent practices – Provide instructions for breast, cervical, and bowel screening, including respecting acceptance or refusal. 3. Address legal and ethical aspects – Link consent to laws and ethical guidance, including situations where capacity is limited. 4. Promote consistency and quality – Use clear, accurate materials so participants and professionals understand the screening process and decision making. | People with intellectual disabilities should continue to receive routine invitations for bowel cancer screening. They may require support from carers or staff to complete the home based FIT/FOBt test kit, as the test is carried out independently at home rather than by a screening practitioner. For individuals who need help to make an informed decision about screening, additional support should be provided. This may include accessible information, verbal explanations, demonstrations, or practical assistance with completing the kit. If a follow-up colonoscopy is required, the person’s capacity to consent should be assessed with appropriate support. Where the individual lacks capacity, decisions should be made in their best interests, following legal and ethical guidance. Recommendations are provided for people with learning difficulties to receive accessible formats including An Easy Guide to Bowel Cancer Screening and An Easy Guide to Having a Colonoscopy |
| 10 | Public Health England | United Kingdom (England) | Bowel (colon / colorectal)⏎Screening | 1. Highlight inequalities – People with learning disabilities participate less in breast, cervical, and bowel screening, leading to poorer outcomes. 2. Identify barriers – Challenges include complex invitations, communication difficulties, mobility issues, lack of support, and misunderstandings by carers or professionals. 3. Emphasize reasonable adjustments – Guidance on adapting invitations, communication, consent, and service delivery to enable informed participation. 4. Support inclusion – Encourage accessible information, inclusive approaches, and adherence to legal duties so disability does not prevent screening. | Provide accessible information and instructions (easy-read leaflets, pictorial guides, videos).- Provide support for carers to help the individual complete tests.- Ensure assistance is available for collecting and returning samples.- Provide practical guidance and reminders to encourage completion.- Continue to invite all eligible PWID and provide follow-up support as needed. Additionally where an individual is considered not to have the capacity to consent, staff involved would need to decide if it’s in the individual’s best interests to be screened. Where the test is non-invasive and painless, such as bowel screening, the decision to proceed would depend on behavioural compliance, as screening is likely to be in the patient’s best interests.” |
| 14 | Leeds Teaching Hospitals NHS Trust | United Kingdom (England) | Bowel (colon / colorectal)⏎Screening | 1. Set a strategic framework – Guide cancer services across prevention, treatment, survivorship, and end-of-life care. 2. Address population needs and inequalities – Integrate prevention and early detection to reduce disparities. 3. Promote quality and person-centred care – Ensure equitable, high-quality care aligned with The Leeds Way values. 4. Support innovation and research – Translate research into improved diagnostics, treatment, and care pathways. 5. Plan service improvements – Strengthen cancer pathway capabilities through partnership and continuous improvement. | The strategy does not list site specific recommendations for breast, cervical, or bowel cancer, but general recommendations, as follows: Collaborate with the Learning Disabilities and Autism Team to make screening information and access equitable. Address barriers to participation through improved support and accessible communication. |
| 13 | Northern Cancer Alliance (NHS partnership) | United Kingdom (England) | Bowel (colon / colorectal)⏎Screening | 1. Highlight low uptake – Notes lower participation in breast, cervical, and bowel screening among people with learning disabilities, contributing to later diagnosis. 2. Provide practical guidance – Offers tools and checklists to understand screening pathways, identify barriers, and make reasonable adjustments. 3. Address consent and capacity – Guides use of the Mental Capacity Act and best interests decision making. 4. Promote equitable access – Supports person-centred approaches to reduce inequalities in early cancer detection. | Bowel Screening (FIT Home Test): • Use a reasonable adjustments checklist and plan. • Provide Easy Read instructions and videos on how to complete the kit. • Offer support from carers to complete the test. • If the person lacks capacity, use best interests decision process. • Document decisions and symptom monitoring in the health action plan. Continuity of care is supported by guidance on next steps following a positive FIT result, including coordination of further investigations and monitoring of signs and symptoms. Bowel Scope Screening (Flexible Sigmoidoscopy): • Use the reasonable adjustments checklist and coordinate with the screening centre. • Provide accessible information and videos to explain the procedure. • Arrange pre-visit and consider sedation options to reduce anxiety. • Make sure all decisions and adjustments are in the health action plan. |
| 11 | North East and Cumbria Learning Disability Network | United Kingdom (England) | Bowel (colon / colorectal)⏎Screening | 1. Document progress – Summarises actions to reduce health inequalities, including improving uptake of breast, cervical, and bowel screening. 2. Highlight initiatives – Describes workstreams, partnerships, training, and materials to support screening access. 3. Identify challenges – Notes ongoing barriers to screening participation and the need for continued action. 4. Support learning and planning – Shares outcomes to inform future planning and replication of effective approaches. | Identification & Flagging: Bowel cancer screening flagging pathway to identify people with learning disabilities within bowel screening hubs, and to trigger additional support through community learning disability teams, and to support informed choice and completion of bowel screening. Accessible Information: Easy Read bowel scope screening guide published nationally; Development of Easy Read FIT kit materials specifically for people with learning disabilities. Workforce Training - Good Bowel Health and Bowel Screening Train-the-Trainer programme, focused on: People with learning disabilities; Families and carers; Supporters and professionals. Identification & Systems • Development of an NHS wide digital alert/flag to support reasonable adjustments Diagnostic Pathways • Reasonably adjusted diagnostic pathways for people with learning disabilities who: o Are referred under the two-week-wait cancer pathway o Require general anaesthetic for diagnostic tests In-patient Screening Access • Specific pathways to enable access to cancer screening for: o People with learning disabilities in secure or inpatient settings o Including adapted call-and-recall processes and workforce training Workforce Education • Targeted training for GPs, practice nurses, screening staff and social care providers focused on: o Cancer screening o Cancer diagnostic pathways o Supporting people with learning disabilities |
| 9 | National Development Team for Inclusion (NDTi), in partnership with the Norah Fry Research Centre (University of Bristol) | United Kingdom (England) | Bowel (colon / colorectal)⏎Screening | 1. Increase screening uptake – Addresses lower participation of people with learning disabilities in cancer screening, which leads to missed opportunities for early detection. 2. Provide practical tools for providers – Offers a strategy and toolkit to help health and care staff support engagement with breast, cervical, and bowel screening by addressing communication, consent, and access barriers. 3. Promote reasonable adjustments – Outlines inclusive practices (e.g. easy-read information, flexible appointments, supported consent) to ensure equitable access. 4. Support local implementation and improvement – Encourages local services to implement, evaluate, and share effective approaches to improve cancer screening participation. | Identification & Follow-up •Local services should identify people with learning disabilities who do not respond to bowel screening invitations. •Active follow-up should be provided rather than reliance on written reminders alone. Communication & Information •Easy-read bowel screening materials should be provided for people with learning disabilities. •Verbal explanations and demonstrations should be offered to support understanding of home based screening kits. Support Mechanisms •Use of screening liaison nurses or learning disability nurses to: •Explain the screening programme. •Demonstrate how to complete the test kit. •Support individuals and carers during decision making. Alternative Diagnostic Approaches •Where standard bowel screening procedures cannot be tolerated; reasonable alternatives may include: •CT colonography •Colonoscopy under general anaesthetic. Involvement of carers or supporters where appropriate Requirement to make reasonable adjustments in anticipation of need |
| 15 | Scottish Government (with NHS and screening partners) | United Kingdom (Scotland) | Bowel (colon / colorectal)⏎Screening | 1. Recognise inequalities in screening uptake - Some groups (e.g. people with disabilities and those experiencing deprivation) are less likely to participate in breast, cervical, and bowel cancer screening, resulting in unequal outcomes. 2. Establish a national equity framework - Set out a strategic vision with priority action areas (communication, access, data, research) to identify and reduce screening inequalities. 3. Guide coordinated implementation and monitoring 4. Define how screening programmes and NHS Boards should work together to improve accessibility, data use, and evaluation so eligible populations benefit equally from early cancer detection. 5. Strengthen evidence and evaluation - Promote better data collection, evidence-based actions, and ongoing review to track progress in reducing inequities across the screening pathway. | All guidance for PWID is general, system level, and applies across all screening programmes: 1. Accessible information and communication: The strategy commits to developing consistent accessible information and communication methods across the screening pathway, to meet the needs of under-served groups, including people with learning disabilities. 2. Engagement with under-served groups: Strengthen engagement with under-served communities and third sector organisations, including co-production of screening information and services. 3. Workforce awareness: Raise awareness of inequalities in screening among screening programme staff and related professionals, to ensure services meet the needs of under-served groups such as people with learning disabilities. 4. Flexible access / reasonable adjustments: Support flexibility of screening appointments (times, locations) and consider reasonable adjustments where needed to improve accessibility for under-served groups, including people with learning disabilities. |
| 14 | Leeds Teaching Hospitals NHS Trust | United Kingdom (England) | Bowel (colon / colorectal)⏎Treatment | 1. Set a strategic framework – Guide cancer services across prevention, treatment, survivorship, and end-of-life care. 2. Address population needs and inequalities – Integrate prevention and early detection to reduce disparities. 3. Promote quality and person-centred care – Ensure equitable, high-quality care aligned with The Leeds Way values. 4. Support innovation and research – Translate research into improved diagnostics, treatment, and care pathways. 5. Plan service improvements – Strengthen cancer pathway capabilities through partnership and continuous improvement. | The strategy does not list site specific recommendations for breast, cervical, or bowel cancer, but a general recommendation, as follows: Work with the Learning Disabilities and Autism Team to ensure equity in treatment access, care delivery, and information provided. |
| 7 | Kinderneurologie.eu | Netherlands | Brain⏎Clinical monitoring | 1. Raise awareness – Cowden syndrome, caused by PTEN mutations, increases the risk of benign and malignant tumors across multiple organs. 2. Highlight relevant cancers and surveillance – People with Cowden syndrome have higher risks of cancers such as thyroid and brain tumors, requiring regular monitoring and early detection. 3. Support clinical decision making – Explains the genetic cause, symptoms, and rationale for syndrome specific cancer screening and follow-up. 4. Guide monitoring and management – Emphasises that, while incurable, early detection of complications including cancer improves outcomes and informs treatment. | People with intellectual disabilities with Cowden syndrome have an increased risk of brain tumors, but the document does not provide any screening, surveillance, or specific guidance for brain tumors. |
| 14 | Leeds Teaching Hospitals NHS Trust | United Kingdom (England) | Breast⏎Prevention | 1. Set a strategic framework – Guide cancer services across prevention, treatment, survivorship, and end-of-life care. 2. Address population needs and inequalities – Integrate prevention and early detection to reduce disparities. 3. Promote quality and person-centred care – Ensure equitable, high-quality care aligned with The Leeds Way values. 4. Support innovation and research – Translate research into improved diagnostics, treatment, and care pathways. 5. Plan service improvements – Strengthen cancer pathway capabilities through partnership and continuous improvement. | The strategy does not list site specific recommendations for breast, cervical, or bowel cancer, but a general recommendation, as follows: Work with the Learning Disabilities and Autism Team to make cancer prevention information equitable and accessible. The strategy identifies reasonable adjustments across cancer services, providing accessible and jargon‑free information for PWID, targeting prevention and screening outreach to the CORE20PLUS population including PWID, ensuring all cancer CSUs complete health‑inequalities training covering learning disability and autism, establishing clear psychological‑support pathways with trained staff, incorporating LD/autism needs into personalised care plans and shared decision‑making, addressing digital exclusion in portals and remote follow‑up, and strengthening partnerships with charities that support PWID. |
| 1 | Belgian Health Care Knowledge Centre (KCE) | Belgium | Breast⏎Screening | 1. Assess disparities in health care access, including use of preventive services. 2. Identify systemic, communication, and organizational barriers. 3. Collect experiences and needs from people with intellectual disabilities, caregivers, and professionals. 4. Develop evidence-based recommendations, including reasonable adjustments, to improve access and equity. | • Tailoring invitations in Easy-to-Read layouts, • Extending appointments for mammograms, • providing clear visual explanations of what screening involves, and • offering support persons during screening appointments. |
| 3 | European Commission, Joint Research Centre (JRC), Directorate F – Health, Consumers & Reference Materials, via the European Commission Initiative on Breast Cancer (ECIBC) | European Union | Breast⏎Screening | 1. Inform screening decision making – Evaluate how targeted versus general communication affects participation, understanding, accessibility, and confidence among women with intellectual disability. 2. Reduce inequalities – Assess whether tailored outreach can increase participation and equity in breast cancer screening for this underserved group. 3. Support evidence-based recommendations – Contribute to ECIBC guidelines using systematic evidence and structured decision frameworks focused on communication strategies. | • Use a targeted communication strategy instead of a general one for invitations to screening to increase participation among women with intellectual disability aged 50–69 (conditional recommendation, low-certainty evidence). • Consider caregiver involvement, as targeted strategies may require extra time or effort from them. • Track participation rates of women with intellectual disabilities in screening. • Evaluate effectiveness of targeted communication strategies. • Support further research on strategies specifically tailored for PWID. |
| 5 | Nederlandse Vereniging van Artsen voor Verstandelijk Gehandicapten (NVAVG) — the Dutch Association of Physicians for People with Intellectual Disabilities | Netherlands | Breast⏎Screening | 1. Clarify risk and applicability – Mammography is effective and women with intellectual disabilities have similar or potentially higher breast cancer risk. 2. Practical and clinical considerations – Addresses challenges such as cooperation, positioning, anxiety, and obtaining quality images. 3. Support clinician decision making – Guides when and how to screen, balancing procedure burden with health benefits. 4. Promote equity in preventive care – Ensures women with intellectual disabilities are included in evidence-based screening and interprets general recommendations for this group. | 1. Support & Assistance o Caregivers should help with positioning during mammography. o Caregivers may assist with completing forms, questionnaires, and explaining the procedure. 2. Consent & Autonomy o Obtain informed consent from the woman whenever possible. o If the woman cannot consent, use legal representative consent. o Do not force the procedure if the woman actively resists. 3. Feasibility / Practical Challenges o Mammography may be difficult or impossible if the woman cannot tolerate it. o Mammogram images may be blurry or incomplete, limiting reliability. 4. Use of Anxiolytic Medication o May be considered for women who are highly anxious or unable to tolerate mammography. 5. Decision to Withhold Further Invitations o If screening is unsuccessful despite maximal support, it is acceptable to stop further invitations. 6. Early Detection Support o Caregivers should monitor breast changes because self-detection may be unreliable. o Manual breast examination is not effective for screening, but caregiver assistance can help detect suspicious changes early. |
| 6 | Stichting Downsyndroom (Down Syndrome Foundation); Vereniging Samenwerkende Ouder- en Patiëntenorganisaties (VSOP); Nederlands Huisartsen Genootschap (NHG) — Dutch College of General Practitioners | Netherlands | Breast⏎Screening | 1. Understand cancer risks – Lists age appropriate screening (cervical, breast, colorectal) and when it should be considered for people with Down syndrome. 2. Recognize screening adaptations – Explains how general prevention programs apply, including necessary adjustments in communication, consent, and follow-up. 3. Integrate into preventive care – Guides GPs to include cancer screening in routine, proactive health management rather than symptom driven care. | Participate in national mammography program Same as general population; no PWID specific adjustments mentioned. |
| 7 | Kinderneurologie.eu | Netherlands | Breast⏎Clinical monitoring | 1. Raise awareness – Cowden syndrome, caused by PTEN mutations, increases the risk of benign and malignant tumors across multiple organs. 2. Highlight relevant cancers and surveillance – People with Cowden syndrome have higher risks of cancers such as thyroid and brain tumors, requiring regular monitoring and early detection. 3. Support clinical decision making – Explains the genetic cause, symptoms, and rationale for syndrome specific cancer screening and follow-up. 4. Guide monitoring and management – Emphasises that, while incurable, early detection of complications including cancer improves outcomes and informs treatment. | Women with Cowden syndrome and intellectual disabilities should undergo a breast examination and breast MRI every year starting at age 25 for early detection of breast cancer. |
| 8 | NHS Cancer Screening Programmes | United Kingdom (England) | Breast⏎Screening | 1. Clarify informed consent – Ensure participation is voluntary and based on understanding screening benefits, risks, and limitations. 2. Guide consent practices – Provide instructions for breast, cervical, and bowel screening, including respecting acceptance or refusal. 3. Address legal and ethical aspects – Link consent to laws and ethical guidance, including situations where capacity is limited. 4. Promote consistency and quality – Use clear, accurate materials so participants and professionals understand the screening process and decision making. | People with learning disabilities should be provided with accessible, Easy Read information about screening and given all practicable support to understand, attend, and participate. Carers or staff may help by explaining the process in an accessible way or offering practical assistance where appropriate. Decisions should be delayed if capacity fluctuates, and if someone cannot decide even with support, a best interests assessment must guide whether screening proceeds. |
| 10 | Public Health England | United Kingdom (England) | Breast⏎Screening | 1. Highlight inequalities – People with learning disabilities participate less in breast, cervical, and bowel screening, leading to poorer outcomes. 2. Identify barriers – Challenges include complex invitations, communication difficulties, mobility issues, lack of support, and misunderstandings by carers or professionals. 3. Emphasize reasonable adjustments – Guidance on adapting invitations, communication, consent, and service delivery to enable informed participation. 4. Support inclusion – Encourage accessible information, inclusive approaches, and adherence to legal duties so disability does not prevent screening. | Provide accessible information about the screening procedure (easy-read, pictorial, videos).- Make every reasonable attempt to obtain satisfactory images, even if positioning is difficult.- Offer practical support and guidance before and during appointments.- Explain alternatives if full mammogram is not possible (e.g., visual breast check).- Provide guidance if consent cannot be given at the time of screening. |
| 14 | Leeds Teaching Hospitals NHS Trust | United Kingdom (England) | Breast⏎Screening | 1. Set a strategic framework – Guide cancer services across prevention, treatment, survivorship, and end-of-life care. 2. Address population needs and inequalities – Integrate prevention and early detection to reduce disparities. 3. Promote quality and person-centred care – Ensure equitable, high-quality care aligned with The Leeds Way values. 4. Support innovation and research – Translate research into improved diagnostics, treatment, and care pathways. 5. Plan service improvements – Strengthen cancer pathway capabilities through partnership and continuous improvement. | The strategy does not list site specific recommendations for breast, cervical, or bowel cancer, but general recommendations, as follows: Collaborate with the Learning Disabilities and Autism Team to make screening information and access equitable. Address barriers to participation through improved support and accessible communication. |
| 13 | Northern Cancer Alliance (NHS partnership) | United Kingdom (England) | Breast⏎Screening | 1. Highlight low uptake – Notes lower participation in breast, cervical, and bowel screening among people with learning disabilities, contributing to later diagnosis. 2. Provide practical guidance – Offers tools and checklists to understand screening pathways, identify barriers, and make reasonable adjustments. 3. Address consent and capacity – Guides use of the Mental Capacity Act and best interests decision making. 4. Promote equitable access – Supports person-centred approaches to reduce inequalities in early cancer detection. | • Use a best interests and reasonable adjustments checklist to plan support. • Provide easy-read information and accessible videos explaining mammography. • Offer pre-visit to meet staff and see equipment before the appointment. • Arrange longer appointment times if needed. • Allow support from carers/family during appointments. • Include screening decisions and adjustments in a health action plan. • If screening is not suitable in best interests, create a breast care plan with symptom monitoring. |
| 11 | North East and Cumbria Learning Disability Network | United Kingdom (England) | Breast⏎Screening | 1. Document progress – Summarises actions to reduce health inequalities, including improving uptake of breast, cervical, and bowel screening. 2. Highlight initiatives – Describes workstreams, partnerships, training, and materials to support screening access. 3. Identify challenges – Notes ongoing barriers to screening participation and the need for continued action. 4. Support learning and planning – Shares outcomes to inform future planning and replication of effective approaches. | Identification & Systems • Screening and clinical system flagging to identify people with learning disabilities and indicate required reasonable adjustments • Development of an NHS wide digital alert/flag to support reasonable adjustments Accessible Information • Easy Read two-week-wait cancer referral information leaflet for people with learning disabilities undergoing urgent cancer investigation Diagnostic Pathways • Reasonably adjusted diagnostic pathways for people with learning disabilities who: o Are referred under the two-week-wait cancer pathway o Require general anaesthetic for diagnostic tests In-patient Screening Access • Specific pathways to enable access to cancer screening for: o People with learning disabilities in secure or inpatient settings o Including adapted call-and-recall processes and workforce training Workforce Education • Targeted training for GPs, practice nurses, screening staff and social care providers focused on: o Cancer screening o Cancer diagnostic pathways o Supporting people with learning disabilities |
| 9 | National Development Team for Inclusion (NDTi), in partnership with the Norah Fry Research Centre (University of Bristol) | United Kingdom (England) | Breast⏎Screening | 1. Increase screening uptake – Addresses lower participation of people with learning disabilities in cancer screening, which leads to missed opportunities for early detection. 2. Provide practical tools for providers – Offers a strategy and toolkit to help health and care staff support engagement with breast, cervical, and bowel screening by addressing communication, consent, and access barriers. 3. Promote reasonable adjustments – Outlines inclusive practices (e.g. easy-read information, flexible appointments, supported consent) to ensure equitable access. 4. Support local implementation and improvement – Encourages local services to implement, evaluate, and share effective approaches to improve cancer screening participation. | Identification & Systems •Breast Screening Systems should record reasonable adjustments required by women with learning disabilities. Communication & Information •Provision of easy-read information for women with learning disabilities. •Use of DVDs, pictorial guides, and photo based materials showing what happens during breast screening. Appointment Structure & Environment •Longer appointments for women with learning disabilities. •Appointments scheduled at quiet times (e.g. first or last of the day). •Offering continuity by arranging the same radiographer where possible. •Provision of appointments at accessible locations, including for wheelchair users. •Screening should be stopped if the woman becomes distressed and re-attempted at a later time. Service Design •Introduction of “disability days”, during which extended appointments and additional support are routinely available for women with disabilities. Inclusion •Women with learning disabilities should remain on breast screening lists and continue to be invited even if they have never attended previously. Involvement of carers or supporters where appropriate Requirement to make reasonable adjustments in anticipation of need |
| 15 | Scottish Government (with NHS and screening partners) | United Kingdom (Scotland) | Breast⏎Screening | • Recognise inequalities in screening uptake Some groups (e.g. people with disabilities and those experiencing deprivation) are less likely to participate in breast, cervical, and bowel cancer screening, resulting in unequal outcomes. • Establish a national equity framework Set out a strategic vision with priority action areas (communication, access, data, research) to identify and reduce screening inequalities. • Guide coordinated implementation and monitoring Define how screening programmes and NHS Boards should work together to improve accessibility, data use, and evaluation so eligible populations benefit equally from early cancer detection. • Strengthen evidence and evaluation Promote better data collection, evidence-based actions, and ongoing review to track progress in reducing inequities across the screening pathway. | All guidance for PWID is general, system level, and applies across all screening programmes: 1. Accessible information and communication: The strategy commits to developing consistent accessible information and communication methods across the screening pathway, to meet the needs of under-served groups, including people with learning disabilities. 2. Engagement with under-served groups: Strengthen engagement with under-served communities and third sector organisations, including co-production of screening information and services. 3. Workforce awareness: Raise awareness of inequalities in screening among screening programme staff and related professionals, to ensure services meet the needs of under-served groups such as people with learning disabilities. 4. Flexible access / reasonable adjustments: Support flexibility of screening appointments (times, locations) and consider reasonable adjustments where needed to improve accessibility for under-served groups, including people with learning disabilities. |
| 16 | Public Health Wales (NHS Wales) | United Kingdom (Wales) | Breast⏎Screening | 1. Encourage participation – Promote attendance at breast screening, including more accessible mobile units. 2. Explain access and support – Provide guidance on contacting centres, arranging appointments, and familiarisation visits. 3. Offer tailored information – Give clear, specific advice to women and carers on how screening can be adapted to individual needs. | Screening location: Mobile units encouraged because they are more accessible. Pre visit / familiarization: Special appointment arrangements can be made to familiarise with staff/equipment/surroundings. Support from carers: Explicitly states women/carers can contact the local screening centre to discuss the invitation and request support. Routine accommodations: Adjustments are offered upon request (e.g., special appointments). |
| 14 | Leeds Teaching Hospitals NHS Trust | United Kingdom (England) | Breast⏎Treatment | 1. Set a strategic framework – Guide cancer services across prevention, treatment, survivorship, and end-of-life care. 2. Address population needs and inequalities – Integrate prevention and early detection to reduce disparities. 3. Promote quality and person-centred care – Ensure equitable, high-quality care aligned with The Leeds Way values. 4. Support innovation and research – Translate research into improved diagnostics, treatment, and care pathways. 5. Plan service improvements – Strengthen cancer pathway capabilities through partnership and continuous improvement. | The strategy does not list site specific recommendations for breast, cervical, or bowel cancer, but a general recommendation as follows: Work with the Learning Disabilities and Autism Team to ensure equity in treatment access, care delivery, and information provided. |
| 14 | Leeds Teaching Hospitals NHS Trust | United Kingdom (England) | Cervix⏎Prevention | 1. Set a strategic framework – Guide cancer services across prevention, treatment, survivorship, and end-of-life care. 2. Address population needs and inequalities – Integrate prevention and early detection to reduce disparities. 3. Promote quality and person-centred care – Ensure equitable, high-quality care aligned with The Leeds Way values. 4. Support innovation and research – Translate research into improved diagnostics, treatment, and care pathways. 5. Plan service improvements – Strengthen cancer pathway capabilities through partnership and continuous improvement. | The strategy does not list site specific recommendations for breast, cervical, or bowel cancer, but a general recommendation, as follows: Work with the Learning Disabilities and Autism Team to make cancer prevention information equitable and accessible. |
| 1 | Belgian Health Care Knowledge Centre (KCE) | Belgium | Cervix⏎Screening | 1. Assess disparities in health care access, including use of preventive services. 2. Identify systemic, communication, and organizational barriers. 3. Collect experiences and needs from people with intellectual disabilities, caregivers, and professionals. 4. Develop evidence-based recommendations, including reasonable adjustments, to improve access and equity. | • tailoring invitations in Easy-to-Read layouts, • extending appointments for Pap tests, • providing clear visual explanations of what screening involves, and • offering support persons during screening appointments. |
| 2 | Region Hovedstaden – the regional health authority for the Capital Region in Denmark | Denmark | Cervix⏎Screening | To inform women with disabilities about the opportunity for HPV Self-Sampling and thereby: 1. Increase screening uptake – Reach women who do not participate in routine cervical screening, especially those for whom clinical exams are difficult. 2. Reduce participation barriers – Enable women with disabilitites to collect samples privately at home. 3. Provide an accessible alternative – Align with national and regional screening strategies; tests can be ordered via a GP and returned by mail. 4. Support early detection and prevention – Facilitate earlier identification of HPV to enable timely follow-up and cancer prevention. | Adjustments • HPV home testing offered instead of clinic based screening • No gynecological examination required • Testing can occur at home or with health professional assistance Guidance • Women with disabilities who cannot take the test themselves should arrange testing with their GP Recommendations • Follow-up examination recommended if HPV positive, in consultation with GP • Participation is voluntary and free of charge |
| 4 | Nederlandse Vereniging van Artsen voor Verstandelijk Gehandicapten (NVAVG) | Netherlands | Cervix⏎Screening | 1. Adapt general standards – Apply the existing Pap smear protocol (every 5 years, ages 30–60) to women with intellectual disabilities. 2. Balance benefit vs. burden – Weigh early detection benefits against physical and psychological challenges of screening. 3. Define eligibility – Identify women for whom screening is appropriate (e.g., sexually active) and those for whom it is not (e.g., never sexually active or refusal). 4. Address practical needs – Consider consent, patient explanation, support persons, sedation, and anxiety reduction during the procedure. | 1. Eligibility & When Not to Screen • Do not offer routine cervical screening to women with intellectual disabilities who, with near certainty, have never had heterosexual sexual contact.* • Do not perform screening if the woman refuses or actively resists, even after explanation. • Do not screen women who have had a hysterectomy involving the cervix (uterine removal). • Do not screen women who have had a satisfactory cervical smear within the past year. 2. Consent & Autonomy • Make sure the woman is well informed about the test before screening. • Obtain informed consent from the woman whenever possible. • If a woman cannot give consent, a legal representative must give consent. 3. Support During the Procedure • Prefer presence of a female caregiver or support person during the screening. • Provide clear explanations of the purpose and steps of the test. • Use visual aids (e.g., pictures) to clarify what will happen. • Allow the woman to see or hold the speculum beforehand to reduce fear. • Use relaxation techniques to help calm the woman during the procedure. • Premedication or sedation may be considered if chosen by the woman. 4. Practical Feasibility • Acknowledge that obtaining an adequate sample may be more difficult in women with intellectual disabilities. • When a smear fails to contain endocervical cells, plan to repeat the smear in 6 months and manage per the usual guideline. |
| 6 | Stichting Downsyndroom (Down Syndrome Foundation); Vereniging Samenwerkende Ouder- en Patiëntenorganisaties (VSOP); Nederlands Huisartsen Genootschap (NHG) — Dutch College of General Practitioners | Netherlands | Cervix⏎Screening | 1. Understand cancer risks – Lists age appropriate screening (cervical, breast, colorectal) and when it should be considered for people with Down syndrome. 2. Recognize screening adaptations – Explains how general prevention programs apply, including necessary adjustments in communication, consent, and follow-up. 3. Integrate into preventive care – Guides GPs to include cancer screening in routine, proactive health management rather than symptom driven care. | Participate in national cervical screening unless sexually inactive Adjustment for PWID: sexually inactive women are exempt; otherwise same as general population. |
| 8 | NHS Cancer Screening Programmes | United Kingdom (England) | Cervix⏎Screening | 1. Clarify informed consent – Ensure participation is voluntary and based on understanding screening benefits, risks, and limitations. 2. Guide consent practices – Provide instructions for breast, cervical, and bowel screening, including respecting acceptance or refusal. 3. Address legal and ethical aspects – Link consent to laws and ethical guidance, including situations where capacity is limited. 4. Promote consistency and quality – Use clear, accurate materials so participants and professionals understand the screening process and decision making. | Women with intellectual disabilities should be invited to the screening programme and supported with accessible information and decision making. If a person cannot consent, a best interests decision should be made before proceeding or removing them from the programme. |
| 10 | Public Health England | United Kingdom (England) | Cervix⏎Screening | 1. Highlight inequalities – People with learning disabilities participate less in breast, cervical, and bowel screening, leading to poorer outcomes. 2. Identify barriers – Challenges include complex invitations, communication difficulties, mobility issues, lack of support, and misunderstandings by carers or professionals. 3. Emphasize reasonable adjustments – Guidance on adapting invitations, communication, consent, and service delivery to enable informed participation. 4. Support inclusion – Encourage accessible information, inclusive approaches, and adherence to legal duties so disability does not prevent screening. | Women with learning disabilities should receive accessible information, practical support at appointments, and help to make an informed decision about cervical screening. If a woman cannot consent, staff must follow the Mental Capacity Act by undertaking a best interests process. Because cervical screening is invasive, this usually requires a formal best interests assessment led by the GP, with support from learning disability specialists, and in line with regional policy. Learning disability alone is never a reason to withhold screening invitations. [2. Cancer…s - GOV.UK \| PDF] |
| 14 | Leeds Teaching Hospitals NHS Trust | United Kingdom (England) | Cervix⏎Screening | 1. Set a strategic framework – Guide cancer services across prevention, treatment, survivorship, and end-of-life care. 2. Address population needs and inequalities – Integrate prevention and early detection to reduce disparities. 3. Promote quality and person-centred care – Ensure equitable, high-quality care aligned with The Leeds Way values. 4. Support innovation and research – Translate research into improved diagnostics, treatment, and care pathways. 5. Plan service improvements – Strengthen cancer pathway capabilities through partnership and continuous improvement. | The strategy does not list site specific recommendations for breast, cervical, or bowel cancer, but general recommendations, as follows: Collaborate with the Learning Disabilities and Autism Team to make screening information and access equitable. - Address barriers to participation through improved support and accessible communication. |
| 13 | Northern Cancer Alliance (NHS partnership) | United Kingdom (England) | Cervix⏎Screening | 1. Highlight low uptake – Notes lower participation in breast, cervical, and bowel screening among people with learning disabilities, contributing to later diagnosis. 2. Provide practical guidance – Offers tools and checklists to understand screening pathways, identify barriers, and make reasonable adjustments. 3. Address consent and capacity – Guides use of the Mental Capacity Act and best interests decision making. 4. Promote equitable access – Supports person-centred approaches to reduce inequalities in early cancer detection. 5. Promotion of dignity sensitive care, alongside the implementation of planned adjustments aimed at minimizing anxiety and distress. | • Use the best interests and reasonable adjustments checklist. • Provide easy read guides and videos about the procedure. • Arrange previsit to reduce anxiety. • Adapt environment (music, familiar items) to comfort the person. • Allow support person to attend. • Consider practice positioning and support for lying comfortably. • PRN antianxiety medication may be arranged if appropriate. • Include plans and symptom awareness in the health action plan. |
| 12 | NHS England | United Kingdom (England) | Cervix⏎Screening | 1. Address inequalities – Tackle lower cervical screening uptake among women with learning disabilities 2. Identify barriers – Highlight challenges in communication, understanding 3. Recommend adjustments – Provide practical ways to adapt invitations, consent, and the screening process. 4. Support informed choice – Promote accessible information and supportive environments to enable participation if desired. | Communication & Understanding • Easy to read information and invitation letters. • Use interpreters, signers, or communication methods the person understands. Preparation & Comfort • Pre visit and familiarisation with equipment. • Use of relaxation methods (music). • Longer/time adapted appointments. • Option to have carer/family present. Consent & Decision Making • Explain what the test does and what it involves. • Ensure understanding of health benefit and ability to consent. • Use best interest decision process when needed. Service/Admin Adjustments • Flagging records for learning disability. • Pre contact before invitation. • Link with learning disability team. • Appointment time communication and double booking. |
| 11 | North East and Cumbria Learning Disability Network | United Kingdom (England) | Cervix⏎Screening | 1. Document progress – Summarises actions to reduce health inequalities, including improving uptake of breast, cervical, and bowel screening. 2. Highlight initiatives – Describes workstreams, partnerships, training, and materials to support screening access. 3. Identify challenges – Notes ongoing barriers to screening participation and the need for continued action. 4. Support learning and planning – Shares outcomes to inform future planning and replication of effective approaches. | Cervical sample taker support pack - Provides information and guidance to professionals to enable effective support of women with learning disabilities in accessing cervical screening. Identification & Systems • Screening and clinical system flagging to identify people with learning disabilities and indicate required reasonable adjustments • Development of an NHS wide digital alert/flag to support reasonable adjustments Accessible Information • Easy Read two-week-wait cancer referral information leaflet for people with learning disabilities undergoing urgent cancer investigation Diagnostic Pathways • Reasonably adjusted diagnostic pathways for people with learning disabilities who: o Are referred under the two-week-wait cancer pathway o Require general anaesthetic for diagnostic tests In-patient Screening Access • Specific pathways to enable access to cancer screening for: o People with learning disabilities in secure or inpatient settings o Including adapted call-and-recall processes and workforce training Workforce Education • Targeted training for GPs, practice nurses, screening staff and social care providers focused on: o Cancer screening o Cancer diagnostic pathways o Supporting people with learning disabilities |
| 9 | National Development Team for Inclusion (NDTi), in partnership with the Norah Fry Research Centre (University of Bristol) | United Kingdom (England) | Cervix⏎Screening | 1. Increase screening uptake – Addresses lower participation of people with learning disabilities in cancer screening, which leads to missed opportunities for early detection. 2. Provide practical tools for providers – Offers a strategy and toolkit to help health and care staff support engagement with breast, cervical, and bowel screening by addressing communication, consent, and access barriers. 3. Promote reasonable adjustments – Outlines inclusive practices (e.g. easy-read information, flexible appointments, supported consent) to ensure equitable access. 4. Support local implementation and improvement – Encourages local services to implement, evaluate, and share effective approaches to improve cancer screening participation. | Identification & Systems •Development of local cervical screening pathways specifically addressing the needs of women with learning disabilities. •Systems should avoid automatic or inappropriate ceasing of women with learning disabilities from cervical screening registers. Communication & Information •Use of easy-read cervical screening information. •Use of visual aids, including anatomy models (e.g. rag doll models), to explain the procedure. Consent & Capacity •Capacity to consent must be assessed where there is doubt. •All practicable steps must be taken to support informed decision making. •Where capacity is lacking, a Best Interests decision making process should be followed. •Decisions about screening participation should not be based on assumptions about sexual activity or tolerance of the procedure. Staff Training •Smear takers should receive training in: •Providing reasonable adjustments for women with learning disabilities. •Applying the Mental Capacity Act in cervical screening contexts. Inclusion •Women with learning disabilities should remain eligible and invited unless a formal decision making process determines otherwise. Involvement of carers or supporters where appropriate Requirement to make reasonable adjustments in anticipation of need |
| 15 | Scottish Government (with NHS and screening partners) | United Kingdom (Scotland) | Cervix⏎Screening | • Recognise inequalities in screening uptake Some groups (e.g. people with disabilities and those experiencing deprivation) are less likely to participate in breast, cervical, and bowel cancer screening, resulting in unequal outcomes. • Establish a national equity framework Set out a strategic vision with priority action areas (communication, access, data, research) to identify and reduce screening inequalities. • Guide coordinated implementation and monitoring Define how screening programmes and NHS Boards should work together to improve accessibility, data use, and evaluation so eligible populations benefit equally from early cancer detection. • Strengthen evidence and evaluation Promote better data collection, evidence-based actions, and ongoing review to track progress in reducing inequities across the screening pathway. | All guidance for PWID is general, system level, and applies across all screening programmes: 1. Accessible information and communication: The strategy commits to developing consistent accessible information and communication methods across the screening pathway, to meet the needs of under-served groups, including people with learning disabilities. 2. Engagement with under-served groups: Strengthen engagement with under-served communities and third sector organisations, including co-production of screening information and services. 3. Workforce awareness: Raise awareness of inequalities in screening among screening programme staff and related professionals, to ensure services meet the needs of under-served groups such as people with learning disabilities. 4. Flexible access / reasonable adjustments: Support flexibility of screening appointments (times, locations) and consider reasonable adjustments where needed to improve accessibility for under-served groups, including people with learning disabilities. |
| 14 | Leeds Teaching Hospitals NHS Trust | United Kingdom (England) | Cervix⏎Treatment | 1. Set a strategic framework – Guide cancer services across prevention, treatment, survivorship, and end-of-life care. 2. Address population needs and inequalities – Integrate prevention and early detection to reduce disparities. 3. Promote quality and person-centred care – Ensure equitable, high-quality care aligned with The Leeds Way values. 4. Support innovation and research – Translate research into improved diagnostics, treatment, and care pathways. 5. Plan service improvements – Strengthen cancer pathway capabilities through partnership and continuous improvement. | The strategy does not list site specific recommendations for breast, cervical, or bowel cancer, but a general recommendation, as follows: Work with the Learning Disabilities and Autism Team to ensure equity in treatment access, care delivery, and information provided. |
| 6 | Stichting Downsyndroom (Down Syndrome Foundation); Vereniging Samenwerkende Ouder- en Patiëntenorganisaties (VSOP); Nederlands Huisartsen Genootschap (NHG) — Dutch College of General Practitioners | Netherlands | Colorectum⏎Screening | 1. Understand cancer risks – Lists age appropriate screening (cervical, breast, colorectal) and when it should be considered for people with Down syndrome. 2. Recognize screening adaptations – Explains how general prevention programs apply, including necessary adjustments in communication, consent, and follow-up. 3. Integrate into preventive care – Guides GPs to include cancer screening in routine, proactive health management rather than symptom driven care. | Participate in national colorectal screening program Same as general population; no PWID specific adjustments mentioned. |
| 7 | Kinderneurologie.eu | Netherlands | Colorectum⏎Clinical monitoring | 1. Raise awareness – Cowden syndrome, caused by PTEN mutations, increases the risk of benign and malignant tumors across multiple organs. 2. Highlight relevant cancers and surveillance – People with Cowden syndrome have higher risks of cancers such as thyroid and brain tumors, requiring regular monitoring and early detection. 3. Support clinical decision making – Explains the genetic cause, symptoms, and rationale for syndrome specific cancer screening and follow-up. 4. Guide monitoring and management – Emphasises that, while incurable, early detection of complications including cancer improves outcomes and informs treatment. | PWID should have a colonoscopy every 5 years starting at age 35 to detect colorectal cancer early. |
| 7 | Kinderneurologie.eu | Netherlands | Kidney⏎Clinical monitoring | 1. Raise awareness – Cowden syndrome, caused by PTEN mutations, increases the risk of benign and malignant tumors across multiple organs. 2. Highlight relevant cancers and surveillance – People with Cowden syndrome have higher risks of cancers such as thyroid and brain tumors, requiring regular monitoring and early detection. 3. Support clinical decision making – Explains the genetic cause, symptoms, and rationale for syndrome specific cancer screening and follow-up. 4. Guide monitoring and management – Emphasises that, while incurable, early detection of complications including cancer improves outcomes and informs treatment. | PWID should undergo a kidney ultrasound every 2 years starting at age 40 to identify renal tumours. |
| 6 | Stichting Downsyndroom (Down Syndrome Foundation); Vereniging Samenwerkende Ouder- en Patiëntenorganisaties (VSOP); Nederlands Huisartsen Genootschap (NHG) — Dutch College of General Practitioners | Netherlands | Leukemia⏎Clinical monitoring | Understand cancer risks – Lists age appropriate screening (cervical, breast, colorectal) and when it should be considered for people with Down syndrome. 2. Recognize screening adaptations – Explains how general prevention programs apply, including necessary adjustments in communication, consent, and follow-up. 3. Integrate into preventive care – Guides GPs to include cancer screening in routine, proactive health management rather than symptom driven care. | Refer to an oncology center if leukemia is suspected Clinical vigilance recommended; no routine screening, but GP must act if symptoms appear. |
| 7 | Kinderneurologie.eu | Netherlands | Skin Melanoma⏎Clinical monitoring | 1. Raise awareness – Cowden syndrome, caused by PTEN mutations, increases the risk of benign and malignant tumors across multiple organs. 2. Highlight relevant cancers and surveillance – People with Cowden syndrome have higher risks of cancers such as thyroid and brain tumors, requiring regular monitoring and early detection. 3. Support clinical decision making – Explains the genetic cause, symptoms, and rationale for syndrome specific cancer screening and follow-up. 4. Guide monitoring and management – Emphasises that, while incurable, early detection of complications including cancer improves outcomes and informs treatment. | PWID should be seen by a dermatologist annually starting at age 30 to check for melanoma or other skin cancers. |
| 6 | Stichting Downsyndroom (Down Syndrome Foundation); Vereniging Samenwerkende Ouder- en Patiëntenorganisaties (VSOP); Nederlands Huisartsen Genootschap (NHG) — Dutch College of General Practitioners | Netherlands | Testicular⏎Clinical monitoring | 1. Understand cancer risks – Lists age appropriate screening (cervical, breast, colorectal) and when it should be considered for people with Down syndrome. 2. Recognize screening adaptations – Explains how general prevention programs apply, including necessary adjustments in communication, consent, and follow-up. 3. Integrate into preventive care – Guides GPs to include cancer screening in routine, proactive health management rather than symptom driven care. | Regular testicular screening by palpation performed by GP/physician Proactive screening due to increased risk in men with Down syndrome; not part of general population screening. |
| 7 | Kinderneurologie.eu | Netherlands | Thyroid⏎Clinical monitoring | 1. Raise awareness – Cowden syndrome, caused by PTEN mutations, increases the risk of benign and malignant tumors across multiple organs. 2. Highlight relevant cancers and surveillance – People with Cowden syndrome have higher risks of cancers such as thyroid and brain tumors, requiring regular monitoring and early detection. 3. Support clinical decision making – Explains the genetic cause, symptoms, and rationale for syndrome specific cancer screening and follow-up. 4. Guide monitoring and management – Emphasises that, while incurable, early detection of complications including cancer improves outcomes and informs treatment. | PWID with Cowden syndrome should have a thyroid ultrasound once a year starting from age 12 to detect any nodules or tumours early. |
| 7 | Kinderneurologie.eu | Netherlands | Uterus (endometrial)⏎Clinical monitoring | 1. Raise awareness – Cowden syndrome, caused by PTEN mutations, increases the risk of benign and malignant tumors across multiple organs. 2. Highlight relevant cancers and surveillance – People with Cowden syndrome have higher risks of cancers such as thyroid and brain tumors, requiring regular monitoring and early detection. 3. Support clinical decision making – Explains the genetic cause, symptoms, and rationale for syndrome specific cancer screening and follow-up. 4. Guide monitoring and management – Emphasises that, while incurable, early detection of complications including cancer improves outcomes and informs treatment. | Women with Cowden syndrome should have a transvaginal ultrasound every year starting at age 30 to monitor for uterine cancer. |

# Supplementary Table 2

**Supplementary Table 2.** AACODS appraisal of the 16 included documents across the six domains (Authority, Accuracy, Coverage, Objectivity, Date, Significance) with the overall count of “Yes” responses (out of 6). Parenthetical qualifiers are the appraisers’ notes. Reproduced verbatim from Vladimir Vukovic’s 1 June 2026 results document.

| Ref | Organisation | Year | Type | Authority | Accuracy | Coverage | Objectivity | Date | Significance | Overall |
| --- | --- | --- | --- | --- | --- | --- | --- | --- | --- | --- |
| 1 | Belgian Health Care Knowledge Centre (KCE) | 2022 | Recommendation | Yes | Yes | Yes | Yes | Yes | Yes | 6/6 |
| 3 | European Commission, Joint Research Centre (JRC), Directorate F – Health, Consumers & Reference Materials, via the European Commission Initiative on Breast Cancer (ECIBC) | 2019 | Recommendation | Yes | Yes | Yes | Yes | Yes | Yes | 6/6 |
| 7 | Kinderneurologie.eu | 2026 | Recommendation (informational/ educational) | Yes | Yes (but difficult since this is an information leaflet) | No | Yes | Yes | Yes | 5/6 |
| 14 | Leeds Teaching Hospitals NHS Trust | 2024 | Policy | Yes | Yes | Yes | Yes | Yes | Yes | 6/6 |
| 9 | National Development Team for Inclusion (NDTi), in partnership with the Norah Fry Research Centre (University of Bristol) | 2013 | Recommendation / practice guidance | Yes | Yes | Yes | Yes | Yes | Yes | 6/6 |
| 4 | Nederlandse Vereniging van Artsen voor Verstandelijk Gehandicapten (NVAVG) | 2003 | Guideline (clinical) | Yes | Yes | Yes | Yes | Yes | Yes | 6/6 |
| 5 | Nederlandse Vereniging van Artsen voor Verstandelijk Gehandicapten (NVAVG) — the Dutch Association of Physicians for People with Intellectual Disabilities | 2004 | Guideline (clinical) | Yes | Yes | Yes | Yes | Yes | Yes | 6/6 |
| 8 | NHS Cancer Screening Programmes | 2009 | Guideline | Yes | Yes | Yes | Yes | Yes (but some procedural recommendation might be outdated) | Yes | 6/6 |
| 12 | NHS England | 2017 (initial), updated 2025 | Recommendation | Yes | Yes (but since it is a recommendation, some of the questions in this section are not applicable) | Not applicable | Yes | Yes | Yes | 5/6 |
| 11 | North East and Cumbria Learning Disability Network | 2017 | Recommendation (informational/educational) | Yes | Yes | No (or not applicable for this type of publication) | Yes | Yes | Yes | 5/6 |
| 13 | Northern Cancer Alliance (NHS partnership) | 2019 | Recommendation | Yes | Yes | Yes | Yes | No | Yes | 5/6 |
| 10 | Public Health England | 2016 | Guideline (practice) | Yes | Yes | Yes | Yes | Yes | Yes | 6/6 |
| 16 | Public Health Wales (NHS Wales) | Not stated | Recommendation | Yes (but no individual authorship) | Yes (does not contain explicit references and methodology) | Yes (but does not provide broader context, evidence) | Yes (but it is service oriented and information and does not discuss alternative perspectives or limitations) | No | Yes (but the webpage provides brief recommendation for service delivery rather than research and analysis) | 5/6 |
| 2 | Region Hovedstaden – the regional health authority for the Capital Region in Denmark | 2023 | Policy | Yes | Yes (but since it is an information leaflet a lot of the questions in this section is not applicable) | No (but not relevant for an information leaflet) | Yes | No | Yes (in the extent that an information leaflet can have impact) | 4/6 |
| 15 | Scottish Government (with NHS and screening partners) | 2023 | Policy | Yes | Yes | Yes | Yes | Yes | Yes | 6/6 |
| 6 | Stichting Downsyndroom (Down Syndrome Foundation); Vereniging Samenwerkende Ouder- en Patiëntenorganisaties (VSOP); Nederlands Huisartsen Genootschap (NHG) — Dutch College of General Practitioners | 2016 | Recommendation | Yes | Yes | Yes | Yes | Yes | Yes | 6/6 |

# Additional policy considerations

*The seven recommendations below are presented as Supplementary “Additional policy considerations”. They remain in the published manuscript and retain full CRediT attribution. The format is identical to the headline shortlist (Recommendations 1–5) in the main report.*

**Recommendation 6 (EU).** The European Commission should invest in the generation and peer-reviewed publication of evidence on intellectual disability specific cancer screening interventions across member states. **Rationale.** The absence of eligible documents from the five academic databases signals a peer-reviewed evidence gap that limits strong EU and national recommendations.

**Recommendation 7 (EU).** The European Commission should address terminological inconsistency between member states as a barrier to policy coherence. **Rationale.** The “learning disability” / “intellectual disability” discrepancy impedes cross-national comparison, data collection, and implementation monitoring, making internationally recognised terminology necessary for EU scoped coherence.

**Recommendation 8 (Member State).** Member states should extend intellectual disability specific cancer policy guidance beyond screening to encompass primary prevention, treatment access, and clinical surveillance. **Rationale.** Prevention specific guidance appeared in only one document and clinical monitoring guidance in only two, both syndrome specific, leaving the continuum beyond screening largely unaddressed.

**Recommendation 9 (Member State).** Member states should establish systematic mechanisms for the registration, dissemination, and periodic review of intellectual disability specific cancer guidance. **Rationale.** The temporal range of included documents and absence of central repositories suggest episodic production without systematic review cycles, risking outdated guidance in practice.

**Recommendation 10 (Programme).** Screening programme commissioners should offer HPV home based self-sampling as a standard alternative pathway for adults with intellectual disability within national cervical screening programmes. **Rationale.** HPV home based self-sampling is a documented feasible alternative for adults unable to tolerate clinic based examination (ref 2), and proactive offering may close a known access gap.

**Recommendation 11 (Programme).** Health system workforce training on the needs of adults with intellectual disability in cancer screening should be formalised and mandated across relevant professional groups. **Rationale.** Workforce training and awareness raising were unmet needs in multiple documents (refs 11, 15), spanning general practitioners, practice nurses, smear takers, radiographers, and screening administrators; training is core policy implementation, not ancillary support.

**Recommendation 12 (Programme).** Health systems should formally recognise and resource the role of carers in intellectual disability cancer screening pathways. **Rationale.** Carers are central to recommended adjustments, from communication support and appointment accompaniment to home based test assistance and symptom monitoring, and screening systems should recognise and resource this role.

# References

1. Ricour C, Desomer A, Dauvrin M, Devos C. How to improve access to health care for people with intellectual disabilities? [Internet]. Brussels: Belgian Health Care Knowledge Centre (KCE). KCE Reports 361; 2022. Available from: https://www.kce.fgov.be doi:10.57598/R361C
2. Region Hovedstaden. Tilbud om HPV-hjemmetest til kvinder med funktionsnedsaettelse [Offer of HPV home tests for women with disabilities] [Internet]. Hoofdstaden, Denmark: Region Hovedstaden; 2025. Available from: https://www.regionh.dk/Sundhed/Screening/Livmoderhalskraeftscreening/Dokumenter/Tilbud%20om%20HPV-hjemmetest%20FINAL-a.pdf
3. European Commission Joint Research Centre (JRC), European Commission Initiative on Breast Cancer (ECIBC). Should a targeted communication strategy vs. a general communication strategy be used for women with intellectual disability? Evidence to Decision (EtD) document [Internet]. Brussels: European Commission; 2019. Available from: https://cancer-screening-and-care.jrc.ec.europa.eu/sites/default/files/Guidelines/EtDs/Updated/ECIBC_GLs_EtD_intellectual_disability_invitation.pdf
4. Nederlandse Vereniging van Artsen voor Verstandelijk Gehandicapten (NVAVG). Standaard 2003-03 Cervixcarcinoom: Screening op cervixcarcinoom bij vrouwen met een verstandelijke handicap [Internet]. Netherlands: NVAVG; 2003. Available from: https://www.nvavg.nl/wp-content/uploads/2014/upload/standaarden/standaard-2003-03-cervixcarcinoom.pdf
5. Nederlandse Vereniging van Artsen voor Verstandelijk Gehandicapten (NVAVG). Standaard 2004-04 Mammacarcinoom: Bevolkingsonderzoek op mammacarcinoom bij vrouwen met een verstandelijke handicap [Internet]. Netherlands: NVAVG; 2004. Available from: https://nvavg.nl/wp-content/uploads/2022/06/standaard-2004-04-mammacarcinoom.pdf
6. Stichting Downsyndroom, Vereniging Samenwerkende Ouder- en Patientenorganisaties (VSOP), Nederlands Huisartsen Genootschap (NHG). Huisartsenbrochure Downsyndroom: informatie voor de huisarts over Downsyndroom [Internet]. Soest: NHG; 2016. Available from: https://www.huisartsengenetica.nl/sites/default/file/Huisartsenbrochure%20Downsyndroom.pdf
7. Kinderneurologie.eu. Cowden syndroom [Internet]. Netherlands: Kinderneurologie.eu; 2026. Available from: https://www.kinderneurologie.eu/ziektebeelden/syndromen/het-cowden-syndroom
8. NHS Cancer Screening Programmes. Consent to Cancer Screening [Internet]. Sheffield: NHS Cancer Screening Programmes; 2009. Available from: https://assets.publishing.service.gov.uk/media/5c126d6040f0b60c8d6019e8/consent_to_cancer_screening.pdf
9. National Development Team for Inclusion, Norah Fry Research Centre. Improving the uptake of screening services by people with learning disabilities across the South West Peninsula – a strategy and toolkit [Internet]. South West Peninsula, UK: NDTi / Norah Fry Research Centre; 2013. Available from: https://www.bristol.ac.uk/media-library/sites/sps/migrated/documents/screeningservices.pdf
10. Public Health England. Cancer screening: making reasonable adjustments for people with learning disabilities [Internet]. London: Public Health England; 2016. Available from: https://www.gov.uk/government/publications/cancer-screening-and-people-with-learning-disabilities/cancer-screening-making-reasonable-adjustments
11. North East and Cumbria Learning Disability Network. Impact Statement 2017–2018: Tackling inequalities for people with a learning disability [Internet]. North East and Cumbria, UK: North East and Cumbria Learning Disability Network; 2018. Available from: https://necldnetwork.co.uk/wp-content/uploads/2021/01/ImpactStatement-20172018-of-the-North-East-and-Cumbria-Learning-Disabil….pdf
12. NHS England. Supporting people with learning disabilities to access cervical screening [Internet]. London: NHS England; 2017. Available from: https://www.gov.uk/government/publications/cervical-screening-supporting-women-with-learning-disabilities/supporting-women-with-learning-disabilities-to-access-cervical-screening
13. Northern Cancer Alliance. Promoting access to cancer screenings for people with a learning disability: guidance and resource pack [Internet]. Northern England, UK: Northern Cancer Alliance; 2019. Available from: https://www.northerncanceralliance.nhs.uk/wp-content/uploads/2019/08/LDN_cancer_pack_2.pdf
14. Leeds Teaching Hospitals NHS Trust. Cancer Care Strategy 2024–2029 [Internet]. Leeds: Leeds Teaching Hospitals NHS Trust; 2024. Available from: https://www.leedsth.nhs.uk/wp-content/uploads/2024/05/Cancer-Care-Strategy.pdf
15. Scottish Government. Scottish Equity in Screening Strategy 2023–2026 [Internet]. Edinburgh: Scottish Government; 2023. Available from: https://www.gov.scot/publications/scottish-equity-screening-strategy-2023-2026/
16. Public Health Wales. Women with a learning disability – breast screening information [Internet]. Wales: Public Health Wales. Available from: https://phw.nhs.wales/services-and-teams/screening/breast-screening/breast-screening-public-information/women-with-a-learning-disability/
